# A genome-wide view of mutation rate co-variation using multivariate analyses

**DOI:** 10.1186/gb-2011-12-3-r27

**Published:** 2011-03-22

**Authors:** Guruprasad Ananda, Francesca Chiaromonte, Kateryna D Makova

**Affiliations:** 1 Center for Medical Genomics, Penn State University, University Park, PA 16802, USA; 2 Integrative Biosciences Program, Penn State University, University Park, PA 16802, USA; 3 Department of Statistics, Penn State University, 505A Wartik Laboratory, University Park, PA 16802, USA; 4 Department of Biology, Penn State University, 305 Wartik Laboratory, University Park, PA 16802, USA

## Abstract

**Background:**

While the abundance of available sequenced genomes has led to many studies of regional heterogeneity in mutation rates, the co-variation among rates of different mutation types remains largely unexplored, hindering a deeper understanding of mutagenesis and genome dynamics. Here, utilizing primate and rodent genomic alignments, we apply two multivariate analysis techniques (principal components and canonical correlations) to investigate the structure of rate co-variation for four mutation types and simultaneously explore the associations with multiple genomic features at different genomic scales and phylogenetic distances.

**Results:**

We observe a consistent, largely linear co-variation among rates of nucleotide substitutions, small insertions and small deletions, with some non-linear associations detected among these rates on chromosome X and near autosomal telomeres. This co-variation appears to be shaped by a common set of genomic features, some previously investigated and some novel to this study (nuclear lamina binding sites, methylated non-CpG sites and nucleosome-free regions). Strong non-linear relationships are also detected among genomic features near the centromeres of large chromosomes. Microsatellite mutability co-varies with other mutation rates at finer scales, but not at 1 Mb, and shows varying degrees of association with genomic features at different scales.

**Conclusions:**

Our results allow us to speculate about the role of different molecular mechanisms, such as replication, recombination, repair and local chromatin environment, in mutagenesis. The software tools developed for our analyses are available through Galaxy, an open-source genomics portal, to facilitate the use of multivariate techniques in future large-scale genomics studies.

## Background

Deciphering the mechanisms of mutagenesis is central to our understanding of evolution and critical for studies of human genetic diseases. The availability of a multitude of sequenced genomes and their alignments provides an opportunity to study mutations on a genome-wide scale in many species, including humans. There is now substantial evidence for within-genome variation in mutation rates; in particular, regional variation in nucleotide substitution rates, insertion and deletion (indel) rates, and microsatellite mutability have been documented across the human genome [1-10]. However, notwithstanding the attention it has received in the literature, the causative mechanisms underlying regional mutation rate variation remain elusive. Biochemical processes, including replication and recombination, have been suggested as potential contributors to mutation rate variation. For instance, replication likely determines the differences in nucleotide substitution rates among chromosomal types - nucleotide substitution rates are highest on chromosome Y, intermediate on autosomes, and lowest on chromosome X (for example, [10,11]), consistent with the relative number of germline cell divisions and thus DNA replication rounds for each of these chromosome types [12,13]. Local male recombination rate has been shown to be a significant determinant of regional nucleotide substitution rate variation [10], supporting the potential mutagenic nature of recombination and/or biased gene conversion [1,6,10]. Rates of small deletions have been found to be associated with replication-related genomic features, and rates of small insertions with recombination-related features [8]. Finally, the role of replication slippage in determining variation in mutability among microsatellite loci has been recently corroborated [9]. Other factors - for example, the predominance of aberrant DNA repair mechanisms like non-homologous end-joining at subtelomeric regions [14], and yet unexplored mutagenic mechanisms potentially acting at telomeres [10] - might influence regional variation in mutation rates as well.

Genome-wide information on three additional genomic features has recently become available. Nuclear lamina binding regions are thought to represent a repressive chromatin environment and are concentrated in the proximity of centromeres [15]; their impact on local mutation rates has not been investigated to date. An abundance of methylated sites at non-CpG DNA locations in human embryonic stem cells was revealed by a recent study [16], suggesting alternative roles for DNA methylation in CpG and non-CpG contexts. Although the function of methylation in generating mutations at CpG locations has been extensively researched [2,6,8-10], no study to date has looked at the potential impact of the non-CpG methylome on the genome and its mutagenesis; in particular, methylated non-CpG cytosines may also elevate mutation rates. Finally, recent predictions of the density of nucleosome-free regions based on MNase digestion [17] can be used to understand the influence of local chromatin structure on mutation rates. Assessing the contribution of these three novel genomic features to mutation rate variation is of obvious and immediate interest.

In addition to varying regionally, rates of different mutations frequently co-vary with each other. Co-variation was observed between rates of nucleotide substitutions (estimated at ancestral repeats and four-fold degenerate sites), large deletions and insertions of transposable elements [2]. In a separate study, co-variation was observed between rates of nucleotide substitutions and both small insertions and small deletions [8]. What causes regional co-variation in the rates of different mutation types? While explanations based on selection have been considered [18], they are not satisfactory because mutation rates also co-vary in presumably neutrally evolving portions of the genome [2]. Shared local genomic landscapes might be responsible for the co-variation of these rates and, on a purely mechanistic basis, one mutation type might be physically associated with another one (for example, indel-induced nucleotide substitutions) [19], causing the corresponding rates to co-vary. However, these hypotheses have never been extensively explored. Notably, while a number of studies have documented regional variation and co-variation of rates of mutations of several types, they have mostly relied on correlation and univariate regression analyses, which relate mutation rates only in a pair-wise fashion, and attempt to explain their variation (as a function of genomic features) one at a time [2,3,5,8-10,18,20-22]. A better understanding of the structure and causes of mutation rate co-variation, which is crucial for studies of mutagenesis, can be achieved only through more sophisticated data analysis approaches.

This is exactly what we pursued in the current study, where we jointly investigated multiple mutation rates alongside several plausible explanatory genomic features, shedding light on the interplay between mutagenesis and the genomic landscape in which it occurs. In more detail, we used multivariate analysis techniques to characterize the co-variation structure of four rates (nucleotide substitutions, insertions, deletions, and microsatellite repeat number alterations) and explore their joint relationship with several genomic landscape variables. First, we applied principal component analysis (PCA) to mutation rates computed along the genome. Next, we linked rates to genomic landscape variables using canonical correlation analysis (CCA). Finally, we applied non-linear versions of these multivariate techniques, kernel-PCA (kPCA) and kernel-CCA (k-CCA), to investigate the presence of non-linear associations. We conducted our analyses on two mutually exclusive neutral subgenomes - one repetitive (ancestral repeats (ARs)) and one unique (non-coding non-repetitive (NCNR) sequences), and three genomic scales (1-Mb, 0.5-Mb, and 0.1-Mb) using human-orangutan comparisons, and repeated them for two additional phylogenetic distances using human-macaque and mouse-rat comparisons, to understand if and how the structure of mutation rate co-variation and the contribution of various genomic features may differ among them.

Importantly, we have made the suite of software tools implemented for this research publicly available, with the aim of improving reproducibility and facilitating future studies of mutation rates and other genome-wide data. We integrated our software into a modular tool set in Galaxy [23], a free and easy-to-use web-based genomics portal that has already established a substantial community of users.

## Results

To investigate co-variation in rates of nucleotide substitutions, small insertions, small deletions, and microsatellite repeat number alterations, we identified all such mutations in the human-orangutan alignments, using macaque as an outgroup to distinguish insertions from deletions. Our rationale for using human-orangutan comparisons is that, since their divergence is greater than that of human and chimpanzee, it is expected to be less affected by biases due to ancestral polymorphisms [24]. We limited our analysis to human-specific mutations occurring after the human-orangutan split in two supposedly neutrally evolving subgenomes; ARs [2] and NCNR sequences [11]. These have been successfully used for evaluating neutral variation in other studies [2,8,10,11,25-27]. Human-specific mutations were chosen because of the high quality of the human genome sequence and its annotation. The AR subgenome consisted of all transposable elements that were inserted in the human genome prior to the human-macaque divergence (thus excluding L1PA1-A7, L1HS, and AluY). The NCNR subgenome was constructed by excluding genes and 5-kb flanking regions around them (thus removing known coding and regulatory elements), other computationally predicted and/or experimentally validated functional elements (see Materials and methods), and all repeats identified by RepeatMasker [28] (excluding mononucleotide microsatellites). This minimizes potential effects of selection and avoids overlap with the AR subgenome.

Next, the human genome was broken into 1-Mb windows, which has been proposed as the natural variation scale for both mammalian nucleotide substitution and indel rates [8,25]. For each 1-Mb window, restricting attention to the AR (and separately NCNR) portion of the window, we computed rates of nucleotide substitutions, small (≤ 30-bp) insertions, small (≤ 30-bp) deletions and mononucleotide microsatellite repeat number alterations (Table 1; see Materials and methods). Moreover, for each 1-Mb window we aggregated genomic features to be used as predictors (Table 2; see Materials and methods). Relationships among mutation rates, and between mutation rates and genomic features, were explored using multivariate analysis techniques, including PCA, CCA, and non-linear versions of both methods. All computations were performed using a suite of tools developed in Galaxy (see Materials and methods).

**Table 1:** Mutation rates investigated in the present study

Type	Measurement	Alignment used
Insertion rate	Insertions/bp	Human-orangutan-macaque
Deletion rate	Deletions/bp	Human-orangutan-macaque
Nucleotide substitution rate	Substitutions/bp	Human-orangutan
Mononucleotide microsatellite mutability	Mutability/bp	Human-orangutan

Mutation rates, which are used as input to PCA and as response set in CCA, are listed, along with the measurement unit and alignments used for their estimation.

**Table 2:** Genomic features investigated in the present study

Feature	Measurement (per Mb)	Source
GC content	Percentage of G and C bases	'GC Percent' track from the UCSC Genome Browser
CpG islands	Count	'CpG island' track from the UCSC Genome Browser
Non-CG methyl-cytosines	Count	[16]
LINE	Count	'RepeatMasker' track from the UCSC Genome Browser
SINE	Count	'RepeatMasker' track from the UCSC Genome Browser
Nuclear lamina	Number of LaminB1 interaction sites with positive intensity	'NKI LaminB1' track from the UCSC Genome Browser
Telomere	Distance in bp	'Gap' track from the UCSC Genome Browser
Female recombination rate (1 Mb)	Centimorgan (cM)	'Recomb rate' track from the UCSC Genome Browser
Male recombination rate (1 Mb)	Centimorgan (cM)	'Recomb rate' track from the UCSC Genome Browser
Recombination rate (0.5 Mb and 0.1 Mb)	Centimorgan (cM)	[82]
SNP	Count	'SNPs 129' track from the UCSC Genome Browser
Replication timing	Time through S-phase	[33]
Nucleosome-free regions	Coverage	[17]
Coding exons	Coverage	'UCSC Genes' track from the UCSC Genome Browser
Conserved elements	Coverage	'28-way most conserved' track from the UCSC Genome Browser

Genomic features, used as predictors in CCA, are listed along with their measurement unit and source. LINE, long interspersed repetitive elements; SINE, short interspersed repetitive element.

To verify whether our findings were consistent over different genomic scales and phylogenetic distances, we produced and analyzed analogous data for the NCNR subgenome considering 0.5-Mb and 0.1-Mb genomic windows, as well as human-macaque alignments (here insertions and deletions were distinguished using marmoset as the outgroup) and mouse-rat alignments (here we studied mouse-specific mutations and distinguished insertions and deletions using guinea pig as the outgroup). Below, we focus on AR and NCNR subgenome results obtained with 1-Mb windows and human-orangutan alignments. Findings for, and comparisons with, other genomic scales/phylogenetic distances analyzed for the NCNR subgenome are provided in the next-to-last subsection of the Results, the Discussion, and in Additional file 1.

### Mutation rate co-variation

**Figure 1:**
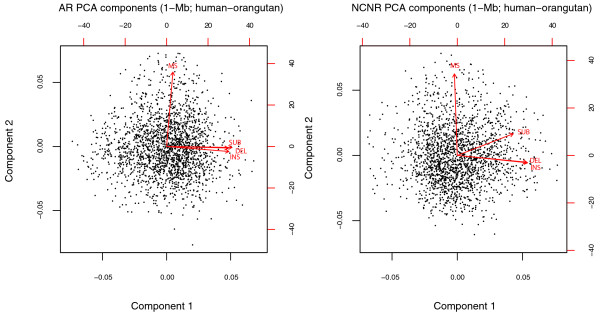
**Biplots of the first two PCA components for our four mutation rates, as obtained from the AR and NCNR subgenomes along the human-orangutan branch for 1-Mb windows**. Black dots represent projected observations (that is, projected windows). The vectors labeled INS, DEL, SUB, and MS depict loadings for insertion rate, deletion rate, substitution rate, and mononucleotide microsatellite mutability, respectively. See Tables S1 and S2 in Additional file 1 for summary statistics.

PCA was used to characterize co-variation among the four mutation rates in terms of orthogonal components, each representing a linear combination of the rates. PCA was run on the correlation matrix (that is, after standardizing the rates) and resulted in two significant components (eigenvalues greater than 1) [29], which accounted for approximately three-quarters of the total variance (Table S1 in Additional file 1). Loadings (eigenvectors), which capture the correlation between each principal component and the rates, were then used to interpret the co-variation structure. Results were largely similar between the AR and NCNR subgenomes (Figure 1).

The first principal component suggested that the strongest co-variation in the genome occurs among insertion, deletion and substitution rates. Insertion and deletion rates exhibited large and concordant loadings for this component in both subgenomes (Figure 1; Table S2 in Additional file 1), indicating a strong positive association between these two mutation rates. Substitution rate also had a large loading for the first principal component in both subgenomes, indicating its association with indel rates.

Microsatellite mutability, which was absent from the first principal component, was the only strong loading in the second principal component in both subgenomes (Figure 1; Table S2 in Additional file 1), suggesting that the variation in this rate is largely orthogonal to the others, and thus that the genomic forces driving microsatellite mutability might be distinct from those driving indel and substitution rates (see below). Interestingly, a marked negative correlation was observed between substitution rates and the number of orthologous microsatellites per 1-Mb window (Figure S1 in Additional file 1). Thus, microsatellite mutability and microsatellite birth/death rates appear to have different dynamics in the genome.

**Figure 2:**
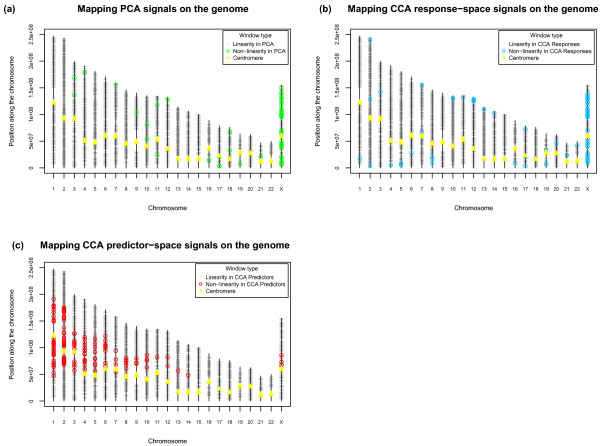
**Genome-wide locations of windows driving non-linear signals in the data**. **(a-c) **Black circles denote windows without marked non-linearity. Green and blue circles denote windows displaying mutation rate non-linearity in PCA (a) and CCA in the response space (b). Red circles denote windows displaying genomic feature non-linearity in CCA in the predictor space (c). Yellow triangles represent the location of the centromeres on each of the chromosomes.

Non-linear relationship between certain mutation types (for example, substitutions and insertions [8]) have been observed by pair-wise comparisons in earlier studies. Investigating non-linear associations (for example, one rate first increasing but then decreasing as another increases; one rate exhibiting more than proportional growth as another increases; one rate 'leveling off' in its growth as another increases) is of interest because they can be suggestive of connections and constraints linking different mutation types. However, questions concerning the strength of such non-linearities, especially when considered as a multiple (as opposed to pair-wise) phenomenon, and whether they tend to occur in particular genomic locations or contexts, have never been addressed directly. To investigate the existence of non-linear associations among multiple mutation rates, we applied kPCA, a variant of PCA that utilizes kernel mapping (see Materials and methods) to compute principal components in a high dimensional space non-linearly related to the original space [30]. While results (Figures S2 and S3 in Additional file 1) were similar to the PCA results described above (with the first principal component dominated by insertion, deletion, and substitution rates, and the second dominated by microsatellite mutability), the scores produced by linear PCA and kPCA for 1-Mb windows, although associated, were not in complete agreement (Figure S4 in Additional file 1). Comparing linear and non-linear PCA scores provides a means to identify genomic regions where neutral mutation rates are co-varying differently from the rest of the genome. We regressed the strongest 'non-linear signal' (scores from the first kernel principal component) onto the 'linear signals' that emerged as significant in the data (scores from the first and second principal components; Table S3 in Additional file 1). The R^2 ^value was 76%, implying that, for the most part, the non-linear signal could be recapitulated by the linear signals. The windows where the non-linear signal was poorly recapitulated by the linear signals were identified as outliers of the regression (see Materials and methods), and a vast majority of them were found to be located either on chromosome X (55% for AR, 64% for NCNR sequences) or at subtelomeric regions of autosomes (Figure 2A; 58% and 45% of autosomal windows in AR and NCNR sequences, respectively, were located within ≤15% of the chromosomal length from the telomeres; see also Figures S5A and S6A in Additional file 1).

### Mutation rate co-variation and genomic landscape

Linking mutation rates and their co-variation to the genomic landscape is crucial for understanding its effects on mutagenesis and thus drawing inferences on potential causal mechanisms. To achieve this, we employed CCA. This is a multivariate technique that, given two sets of variables (for example, responses and predictors), extracts pairs of components (each comprising a linear combination in the response space, and a linear combination in the predictor space) that are maximally correlated to one another - like PCA, subsequent pairs have orthogonal response components, and orthogonal predictor components [31]. This provides a way of simultaneously associating multiple mutation rates (responses, Table 1) to multiple genomic features (predictors, Table 2).

We used the four mutation rates introduced above as our response set, and formed a predictor set that included genomic features shown to associate with mutation rates in previous studies (GC content, recombination rates, number of CpG islands, proximity to telomere, replication timing, number of long interspersed repetitive elements (LINEs), number of short interspersed repetitive element (SINEs), density of SNPs, density of coding exons and density of conserved elements) [2,5,6,8-10], as well as features not formerly considered (number of nuclear lamina binding sites, abundance of non-CG methyl-cytosines, and density of nucleosome-free regions; Table 2). Some of these genomic features are correlated (for example, GC content and replication timing [32,33]), and one can investigate their co-variation structure through PCA as was done for the mutation rates (PCA results for genomic features are reported in Figure S7 and Tables S4 and S5 in Additional file 1). However, our focus here is not on identifying leading components of the local variation in genomic landscape, but rather leading components of its effects on mutation rates - to this end, extracting CCA components is more effective and easier to interpret than correlating principal components extracted separately for mutation rates and genomic features.

CCA yielded four canonical component pairs in the NCNR subgenome and four in the AR subgenome. The correlations observed for these pairs were 0.6955, 0.5043, 0.3906 and 0.1043 for the NCNR subgenome, and 0.7338, 0.5336, 0.3287 and 0.0534 for the AR subgenome. Based on *P*-values from Rao's F Approximation test [34] (see Materials and methods), all four NCNR pairs and the first three AR pairs were significant (*P*-values < 2.2e-16, < 2.2e-16, < 2.2e-16, and 0.0116 for NCNR, and < 2e-16, < 2e-16, < 2e-16, and 0.7637 for AR; Table S6 in Additional file 1). Remarkably, the first three AR and NCNR response components described very similar patterns (although differing in order; see below). Loadings, which capture the correlations between canonical components belonging to each pair and the rates (in the response space) or the genomic features (in the predictor space), were then used for interpretation.

**Figure 3:**
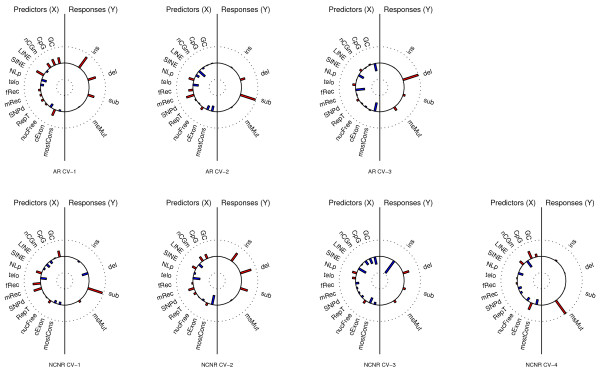
**Helioplots for CCA performed on the AR and NCNR sub-genomes along the human-orangutan branch for 1-Mb windows**. The labels on the plots are as follows: CV, canonical variate; GC, GC content; CpG, number of CpG islands; nCGm, number of non-CpG methyl-cytosines; LINE, number of LINE elements; SINE, number of SINE elements; NLp, number of nuclear lamina associated regions; telo, distance to the telomere; fRec and mRec, female and male recombination rates; SNPd, SNP density; RepT, replication time; nucFree, density of nucleosome-free regions; cExon, coverage by coding exons; mostCons, coverage by most conserved elements. Red bars indicate positive loadings, and blue bars negative loadings. See Table S6 in Additional file 1 for summary statistics.

The first AR response component and the second NCNR response component were very similar to one another (and similar to the first principal component); they showed strong and concordant loadings for insertion rates, deletion rates and substitution rates (Figure 3). Thus, these components render a direction of strong co-variation for indel and substitution rates. The corresponding predictor components in both subgenomes showed strong loadings for GC content, number of CpG islands, non-CpG methylated sites, SINEs and density of coding exons (all displaying a positive association with the responses), as well as number of nuclear lamina binding sites and density of nucleosome-free regions (both negatively associated with the responses). Therefore, the first AR and second NCNR canonical component pairs suggest that nucleosome-free regions with many nuclear lamina binding sites, low GC content, fewer SINEs and fewer coding exons are less prone to insertions, deletions and nucleotide substitutions (Figure 3). Male recombination rate (positively associated with the responses), as well as distance from telomere and density of conserved elements (both negatively associated with the responses) appear alongside all of the above-mentioned genomic features as strong contributors to the second NCNR predictor component.

The second AR response component and the first NCNR response component were similar to one another, and both had dominant nucleotide substitution rate loadings (Figure 3). Thus, these components render a direction of strong nucleotide substitution rate variation. The corresponding predictor components in both subgenomes had strong positive loadings for recombination rates, and strong negative loadings for distance to telomere. The predictor component in the NCNR subgenome also had a strong positive loading for GC content.

The third AR and NCNR response components showed strong loadings for deletion rates (Figure 3). In addition, the NCNR component also displayed a strong loading for insertion rates. Thus, these components render a direction of deletion rate variation in both subgenomes, additionally depicting a negative co-variation between indel rates in the NCNR subgenome. In both subgenomes, the corresponding predictor component had negative loadings for GC content, female recombination rate, SINE counts, and density of conserved elements. Additionally, in the NCNR subgenome, the third predictor component had sizeable positive loadings for density of nucleosome-free regions, and negative loadings for density of coding exons.

Finally, although not significant in the AR subgenome, the fourth response components in both the AR and NCNR subgenomes had dominant microsatellite mutability loadings (Figure 3). Thus, these components render a direction of strong microsatellite mutation rate variation. The marginal correlations between these and the corresponding predictor components (0.104 and still significant in NCNR, 0.053 and non-significant in AR), and the smaller number of predictors with sizeable loadings, confirm a lesser role of genome landscape features in explaining microsatellite mutability [9]. Nevertheless, it is important to note a positive association between microsatellite mutability and the density of CpG islands, and a negative association between microsatellite mutability and counts of methylated non-CpG sites.

Non-linear relationships between mutation rates and genomic landscape variables have been noted in previous studies, and usually investigated through pair-wise comparisons (for example, biphasic effect of GC content on substitution rates [10]). Investigating non-linear associations between mutations and genomic context can provide crucial insights into mutagenesis mechanism. Here, we are interested in detecting and interpreting non-linear signals linking multiple mutation rates to multiple genomic features, and on locating these signals along the genome. We applied kCCA, a variant of CCA that uses kernel mapping to compute canonical components in high dimensional spaces non-linearly related to response and predictor spaces [35]. Plotting linear CCA and kCCA scores against one another (Figure S8 in Additional file 1) suggested non-linearity in the association of mutation rates to the genomic landscape, comprising a small non-linearity in mutation rates, and a more noticeable one in genomic features. To further explore this, we regressed the strongest 'non-linear signals' in response and predictor space (scores from the first kernel CCA response and predictor components) onto significant 'linear signals' (scores from significant linear CCA response and predictor components; Table S7 in Additional file 1). For the response space (mutation rates), the dominant non-linear signal was almost entirely recapitulated by the significant linear signals (R^2 ^higher than 99% for both AR and NCNR sequences). However, for the predictor space (genomic features), significant linear signals could account for merely 1% of the variance of the dominant non-linear signal. Thus, when considering signals associating mutation rates and genomic landscape features, non-linearities displayed by the latter are much stronger than those displayed by the former.

We again used outliers from the regressions to identify genomic locations 'driving' non-linearity in mutation rates and genomic features - that is, windows for which non-linear signals were poorly recapitulated by linear ones (see Materials and methods). In the case of the responses, non-linearity was minimal (R^2 ^above 99%; Table S7 in Additional file 1), but, interestingly, results paralleled those obtained with PCA signals. The majority of outlying loci were on chromosome X (64% for AR - Figure S5B in Additional file 1; 52% for NCNR sequences - Figure S6B in Additional file 1) or near autosomal telomeres (Figure 2B; 42% and 62% of autosomal windows in AR and NCNR sequences, respectively, were located within a distance ≤10% of the chromosomal length from the telomeres; see also Figures S5B and S6B in Additional file 1). These are regions of the genome where mutation rates are sizably lower (chromosome X) or higher (telomeres) than autosomal averages. In the case of the genomic features, the non-linearity was very marked (R^2 ^of merely 1%; Table S7 in Additional file 1), and a vast majority of the loci driving this strong non-linearity were concentrated around the centromeres of large chromosomes (Figure 2C; 49% and 51% of such windows in AR and NCNR sequences, respectively, were within a distance of ≤15% of the chromosomal length from the centromere; see also Figures S5C and 6C in Additional file 1).

### Consistency across genomic scales and phylogenetic distances

To verify whether our findings could be reproduced over different genomic scales and phylogenetic distances, in addition to the 1-Mb windows and human-orangutan comparison investigated above, we repeated our analyses considering 0.5-Mb and 0.1-Mb genomic windows as well as human-macaque and mouse-rat comparisons. Interestingly, the mutation rate co-variation structure remained largely consistent across all three genomic scales and all three phylogenetic distances (Figure 1; Figures S9 to S17 in Additional file 1). Nevertheless, we did observe some differences. For instance, while microsatellite mutability varied orthogonally to indel and substitution rates at the 1-Mb scale, a co-variation (at best moderate) linking microsatellite mutability to the three rates was shown by PCA at smaller scales (0.5 Mb and 0.1 Mb). CCA results also captured this co-variation, with SINE counts and GC content being the major contributors (both negative; Figures S13 to S16 in Additional file 1). Considering multiple window sizes also provided insights into the scale at which various genomic features affect the structure of mutation rate co-variation. For instance, replication timing, SNP density and density of nucleosome-free regions become significant predictors of microsatellite mutability at smaller scales (Figures S13 to S17 in Additional file 1). These associations are noted here for the first time, as previous studies only considered microsatellite mutability at scales of 1 Mb or larger [9]. Further, the association of mutation rates with genomic features showed some differences between the rodent branch and the two primate branches (Figure S17 in Additional file 1). For instance, the effect of recombination on mutation rates was found to be substantial in the primate comparisons, and barely marginal in the rodent comparison. Such differences are expected given the fact that primates and rodents are known to differ in both genomic landscape characteristics and mutation rates [36].

### Toolset in Galaxy

**Figure 4:**
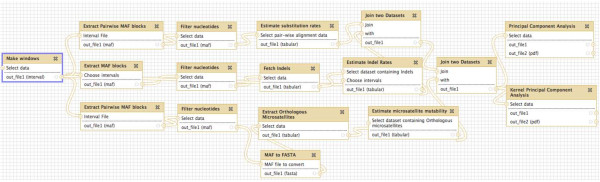
**Galaxy workflow developed for estimating mutation rates and computing principal components**. A similar workflow (not shown) was implemented to compute canonical correlation component pairs. MAF, multiple alignment format.

Comparative genomic studies like ours often process enormous amounts of sequence and alignment data, the storing and handling of which poses big challenges. Having data and software tools on a single platform can substantially facilitate genome-wide analyses and improve reproducibility of results (see, for instance, a workflow for the present study in Figure 4). To disseminate the software developed for our project to the research community, we used Galaxy [23] - a free, open-source genomics portal with a consistent and easy-to-use interface capable of handling vast amounts of data. Galaxy stores all sequences and alignments locally, and provides a multitude of software tools organized in different sections. The ones we developed (Table 3) are available under the 'Regional variation', 'Multiple regression', and 'Multivariate analysis' sections, and include software for alignment data preprocessing, identification of mutations and computation of rates, aggregation of genomic variables, and statistical analyses (more details are provided in the Materials and methods).

**Table 3:** 'Regional variation', 'multiple regression' and 'multivariate analysis' toolsets in Galaxy

Data pre-processing tools	
Make windows	To partition genome into windows of a user-specified size
Feature coverage	To apportion various genomic features in genomic windows
Filter nucleotides	To identify and mask low-quality nucleotides from alignments based on a quality score cutoff specified by the user
Mask CpG/non-CpG sites	To identify and mask CpG/non-CpG-containing sites from alignments
Tools for identifying mutations and computing their rates	
Fetch Indels	To identify insertions and deletions from three-way alignments using a user-specified outgroup
Estimate indel rates	To estimate indel rates by aggregating insertions and deletions in genomic regions specified by the user
Fetch substitutions	To identify nucleotide substitutions from pair-wise alignments
Estimate substitution rates	To estimate substitution rate according to Jukes-Cantor JC69 model
Extract orthologous microsatellites	To fetch microsatellites using SPUTNIK, and detect orthologous repeats
Estimate microsatellite mutability	To estimate microsatellite mutability by grouping (and sub-grouping) repeats based on their size, unit and motif
Multiple regression tools	
Perform linear regression	To construct a linear regression model using the user-selected predictors and response variables
Perform best-subsets regression	To examine all of the linear regression models that can be created from all possible combinations of the predictors variables
Compute RCVE	To compute RCVE (relative contribution to variance) for all possible variable subsets
Multivariate analysis tools	
PCA	To perform PCA on a set of variables
CCA	To perform CCA on two sets of variables
Kernel PCA	To perform kernel PCA on a set of variables, using a user-specified kernel
Kernel CCA	To perform kernel CCA on two sets of variables, using a user-specified kernel

RCVE, relative contribution to variability explained.

## Discussion

In this study we investigate regional co-variation among mutation rates in largely neutrally evolving parts of the human genome (the AR and NCNR subgenomes), and its association with features of the genomic landscape. For the first time, the structure and causes of mutation rate co-variation were studied via a multivariate approach considering several mutation types and a large number of genomic features jointly. Notably, the similarity in results obtained for the AR and NCNR subgenomes lends support to the notion of a common denominator shaping mutagenesis in both repetitive and unique parts of the genome.

### Association of insertion, deletion and substitution rates, and its causes

As indicated by the first principal component of our PCA analysis, the strongest co-variation in the genome is among insertion, deletion, and substitution rates. While this association has been suggested by previous pair-wise analyses [8,37], here we are able to speculate about its causes using the CCA results. The first AR and second NCNR canonical component pairs (Figure 3) suggest that the co-variation of indel and substitution rates is shaped by a common set of genomic features. Some of these features have been found to affect rates of individual mutation types in previous studies; in particular, GC content, number of CpG islands and SINEs, and density of coding exons have been shown to associate positively with indel rate and substitution rate variation [2,5,8,10]. Other genomic features are investigated here for the first time; we show that non-CpG methyl-cytosines, nuclear lamina binding sites and nucleosome-free regions are significant contributors to mutation rate co-variation, suggesting a role for non-CpG methylation, nuclear lamina association, and chromatin structure in mutagenesis.

The positive effect of GC content, density of coding exons and non-CpG methyl-cytosines on mutation rates underlines the role of methylation in creating mutation hotspots [38,39], while the negative effect of number of nuclear lamina binding sites and density of nucleosome-free regions suggests that regions associated with the lamina and/or having compact chromatin structures are less prone to mutations. Distance from telomere appears alongside all of the above mentioned genomic features as a strong contributor to the second NCNR predictor canonical component, with a negative association with the responses, which emphasizes peculiar mutagenic mechanisms acting near telomeres [6,8,10,40]. Notably, the number of nuclear lamina binding sites is positively associated with the distance to telomere in this component; in agreement with another study [15], this indicates that lamina binding regions might be less mutable when they are located at a distance from the telomeres.

The first AR and second NCNR canonical component pairs suggest that genomic regions with many nuclear lamina binding sites, a high density of nucleosome-free regions, low GC content, low exon density, and fewer SINEs are less prone to insertions, deletions and nucleotide substitutions (Figure 3). Regions associated with nuclear lamina constitute a strongly repressive chromatin environment [15], low-GC and gene-poor regions are known to possess compact chromatin structure and higher concentration of indels [41-43], and the preferential retention of SINEs in GC-rich regions has also been linked to the chromatin structure (SINE integration may be facilitated by chromatin decondensation in GC-rich regions) [44]. Further, these component pairs show the density of nucleosome-free regions to be positively associated with nuclear lamina counts, and negatively associated with both GC content, density of CpG islands and coding exons. In all, the picture is one of nucleosome-free regions characterized by a compact chromatin structure.

In summary, the first AR and second NCNR CCA component pairs suggest that methylation and chromatin structure may have a dominant role in the strong co-variation of indel rates and substitution rate - typifying an inverse relationship between compact chromatin structure and proneness of DNA to indels and substitutions. This can perhaps be attributed to the low rate of lesion formation in compact chromatin regions [45] and to the differences in repair mechanisms between different chromatin environments [46].

The third AR and NCNR CCA component pairs depict deletion rate variation, with the third NCNR CCA component pairs also indicating a negative association between insertion and deletion rates (Figure 3). The corresponding predictor components have negative loadings for GC content, SINE counts and density of conserved elements (the latter only for the AR subgenome). GC-poor regions are known to be late-replicating [32,33] and more prone to replication errors [47], which accounts for the elevated mutation rates; our observation therefore supports a role of replication in generating deletions. Furthermore, we confirm the negative association between SINE counts and deletion rates observed previously [8,21]. The positive association of GC content and density of coding exons with insertion rates, and their negative association with deletion rates, point to genomic regions that tolerate more insertions than deletions; such regions were indeed found to be present in GC-rich, gene-rich isochores in Venter's genome by a recent study [43]. The negative association of the density of conserved elements with deletion rates reiterates a previous observation about conserved and functional regions being depleted of small deletions [8].

A set of features comprising male and female recombination rates and distance to telomere was identified as affecting substitution rates through the second AR and the first NCNR CCA component pair (Figure 3). These again reflect the role of recombination in contributing to substitution rate variation [1,2,6,10,48], and reiterate the presence of mutagenic mechanisms acting near telomeres that can lead to elevated nucleotide substitution rates [10]. Alternatively, or additionally, telomeres might possess fixation biases, for example, due to biased gene conversion [49]. The strong positive loading for GC content in the NCNR subgenome is a possible consequence of recombination-associated mismatch repair, which is GC-biased in mammals [48,50,51].

### Microsatellite mutability and its genomic determinants

Our results suggest that microsatellite mutability is driven by different factors than indel and substitution rates. Indeed, microsatellite mutability was the only significant contributor to the second PCA component, indicating a variation largely orthogonal to that of the other three mutation rates. No association between microsatellite mutability (computed here for mononucleotide microsatellites only) and substitution rate was found also in another recent study [9]. The presence of a negative correlation between microsatellite density and substitution rates (Figure S1 in Additional file 1) confirms the findings of Zhu and colleagues [52], and suggests differences in the dynamics and genomic landscape correlates of microsatellite mutability and microsatellite birth-death frequency.

The fourth AR and NCNR CCA component pairs, which are dominated by microsatellite mutability on the response side, are characterized by marginal correlations and smaller numbers of genomic features with sizeable loadings, suggesting an insubstantial role of the genomic landscape in explaining microsatellite mutability. In agreement with this, a recent study concluded that a microsatellite's intrinsic features (repeat number, length and motif identity) are the primary determinants of its mutability [9]. Nevertheless, it is important to note a positive association between microsatellite mutability and the density of CpG islands, and a negative association between microsatellite mutability and counts of methylated non-CpG sites. Together, these observations suggest that microsatellite mutability is suppressed in methylated regions (CpG islands are usually unmethylated) [39].

### Nonlinear trends in mutation rate co-variation and its relationship with genomic predictors

A comparison of scores from PCA and kPCA indicates some departure from linearity in the mutation rate co-variation structure. Non-linearities in the relationship between insertion and deletion rates, as well as between indel and substitution rates, have been noted earlier [8]. However, previous analyses were only pair-wise (that is, did not consider several mutation types simultaneously) and did not focus on identifying regions responsible for non-linear signal. Here we show that genomic loci driving non-linearities in mutation rate co-variation are concentrated on chromosome X and in proximity to the telomeres of autosomes, suggesting a role for the unique landscape of chromosome X, as well as unexplored mutagenic mechanisms acting near telomeres. Indeed, loci with the strongest departures from linearity tend to concentrate in those parts of the genome where rates of nucleotide substitutions, insertions and deletions are markedly higher (telomeres) or lower (chromosome X) than the corresponding autosomal averages [4,6,8,10,23,40,53]. In comparison with autosomes, chromosome X is GC-poor [54,55], late replicating [33] and has a biased distribution of LINE and SINE transposable elements [26,56-58], while autosomal subtelomeric regions are relatively GC-rich [6], have higher recombination rates [6,59-61] and are enriched for double-stranded breaks/repair [14,62]. Such differences in genomic landscape features might indeed substantially impact the structure of mutation rate co-variation.

In contrast, a comparison of scores from CCA and kCCA stressed departures from linearity on the predictor side (genomic features), with genomic loci driving non-linearities mostly concentrated around the centromeres of large autosomes. This suggests that chromosome size, unexplored mutagenic mechanisms acting near centromeres, and other factors (for example, repair differences between subtelomeric regions and other parts of the genome) may be responsible for non-linear signals associating mutation rates and genomic landscape features. Indeed, the non-linearities we detected through kCCA, although much more marked for genomic features, may recapitulate non-linear trends observed pair-wise in previous studies; for example, non-linear relationships between nucleotide substitution rates and GC content [2,6,10,63], indel rates and GC content [8], substitution rates and distance to telomere [10], and insertion rates and distance to telomere [8]. A possible interpretation of the high concentration of 'non-linearity driving' loci around the centromeres of large chromosomes is that non-linear signals might manifest themselves only at a sufficient distance from the telomeres (an average absolute distance of at least 60 Mb), with smaller chromosomes devoid of such loci because this distance cannot be achieved. Other interpretations could involve differences between subtelomeric regions [14] and regions away from the telomeres [62] relative to DNA repair.

In summary, we uncovered important information on how a shared local genomic landscape shapes the co-variation structure of mutation rates. The landscape surrounding centromeres of large autosomes comprises strong non-linearities among genomic predictors as they affect mutation rates - correspondingly, the latter are linearly related and have moderate values. Subtelomeric and chromosome X landscapes differ notably, with genomic predictors behaving linearly as they affect mutation rates, and the latter showing non-linearities and extreme values (high and low, respectively). Interestingly, the landscape throughout small autosomes appears similar to the subtelomeric landscape of larger autosomes, suggesting that a region must be sufficiently removed from telomeres in absolute terms before non-linearities among genomic predictors can occur. We note that a similar landscape (with genomic features behaving linearly as they affect mutation rates) on small autosomes might stem from the spatial proximity and preferential interactions among these chromosomes [64].

### Results at finer genomic scales

Results obtained repeating our analyses with 0.5-Mb and 0.1-Mb windows largely agreed with those described and discussed for 1-Mb windows. In particular, the co-variation between indel and substitution rates was observed and found to be dictated by a common set of genomic features at all three scales (Figures 1 and 3; Figures S9 to S17 in Additional file 1). Similarly, substitution rate variation was also observed at all three scales and found to be driven by the same genomic landscape features. However, some differences were observed with respect to microsatellite mutability. Unlike at the 1-Mb scale, at finer scales microsatellite mutability did show some co-variation with indel and substitution rates. In addition, genomic features appeared to have a stronger effect on microsatellite mutability at finer scales. While at the 1-Mb scale only CpG islands and methylated non-CpG sites showed a (mild) association with microsatellite mutability, at the 0.5-Mb and 0.1-Mb scales density of nucleosome-free regions, density of SNPs and replication timing were also found to be significant predictors (Figure 3; Figures S12 to S17 in Additional file 1). This hints to a possible role of microsatellites in attracting SNPs in their neighborhood (positive loading for SNP density), likely facilitated by an interaction between heterozygous sites and mismatch repair process [65-67], which is known to be less effective in late replicating regions [68] (positive loading for replication timing). These findings are evidence that genomic landscape effects on microsatellite mutability cannot be completely disregarded. When observed at larger scales (for example, 1-Mb and 5-Mb, as seen here and in [9]) microsatellite mutability appears mostly driven by their intrinsic features. However, when focusing on finer scales (0.5 Mb and 0.1 Mb), genomic landscape features seem to gain significant influence. It is important to remark that these observations should be considered preliminary because we only analyzed mononucleotide microsatellites; higher order microsatellites (di-, tri-, tetra-nucleotide microsatellites) are known to have very different dynamics, and may show distinct associations with the genomic landscape.

### Results for the human-macaque and mouse-rat comparisons

Both the structure of mutation rate co-variation and its association with the genomic landscape appear similar in human-orangutan and human-macaque comparisons (Figures 1 and 3; Figures S9 to S16 in Additional file 1), which span rather different evolutionary distances (approximately 12 million years and approximately 25 million years, respectively). While other phylogenetic distances should be considered in other studies, this suggests that our results are not dictated by specific evolutionary distances.

The structure of mutation rate co-variation in the mouse-rat comparison (approximately 12 to 24 million years) was found to be very similar to that on the primate branches (Figures 1 and 3; Figures S9 to S17 in Additional file 1). However, the association of mutation rates with certain genomic landscape variables seemed to differ - in particular, GC content, SNP density, recombination rates, and LINE and SINE counts (Figure S17 in Additional file 1). This could be attributed to differences between the genomic landscapes of primates and rodents; specifically, when compared to the human genome, the mouse genome has higher mean GC content, more active L1 elements, lower overall levels of recombination, and higher mutation rates [36,59]. Recombination rates, which appeared to significantly influence indel and substitution rates in human-orangutan and human-macaque comparisons, showed barely minimal influence in the mouse-rat comparison, consistent with previous observations that the role of recombination rate in rodent mutagenesis is at best moderate [59,69].

## Conclusions

The use of multivariate techniques was crucial to our investigation of mutation rate co-variation and its relationship with the genomic landscape, as it allowed us to consider several rates and several genomic features simultaneously. The important insights we were able to gather regarding mammalian mutagenesis could previously only be speculated about indirectly, if at all, through pair-wise and univariate analyses. Moreover, our *in silico *results provide useful hypotheses (for example, the decoupling of microsatellite and indel/substitution mutations and their contrasting relationship with the underlying genomic landscape; the likely role of non-CpG sites and nuclear lamina binding regions in mutagenesis) that can be further evaluated in wet-lab experiments (see, for instance, the hybrid computational/wet-lab approach we adopted in [70]).

In addition to an improved understanding of mutagenesis, our work has direct application to related areas of genomic research, such as the prediction of functional elements. Identification of functional elements in non-coding regions of the genome is contingent upon the ability to clearly discriminate functional sites from neutrally evolving ones, which is complicated by regional variation of neutral mutation rates. Previous studies have indicated that conservation, or more generally alignment-based scores, have increased performance in the prediction of functional elements when corrected to incorporate local substitution rates [10,71,72]. Future studies could employ our results when designing local background corrections in prediction algorithms. The significant signals obtained from our principal component and canonical correlation analyses could be used as composite correction variables, taking into account simultaneously multiple mutation types and/or genome landscape features. Notably, the results of our kernel principal components and canonical correlation analyses suggest that, while in some regions of the genome linear composites of mutation rates and/or landscape features will be satisfactory, non-linear composites may be much more effective in others.

The statistical and computational tools developed for our study have been integrated into Galaxy, a user-friendly genomics platform [23]. Our multivariate analysis tools are therefore available to the scientific community to reproduce our results, to investigate mutation rate co-variation in other genomes, and to address a plethora of other important biological questions on a genome-wide scale.

## Materials and methods

### Data acquisition and pre-processing

Two types of presumably neutrally evolving subgenomes, the NCNR subgenome and the AR subgenome, were constructed based on the March 2006 build of the human genome (hg18). The NCNR subgenome was constructed by excluding known genes (and the 5-kb flanking regions surrounding them) as annotated at the UCSC Genome Browser [73-75] and known functional elements, including experimentally validated ones (CTCF binding sites, estrogen receptor binding sites, RNA polymerase II binding sites), and computationally predicted ones (most conserved elements produced by phastCons, vista enhancers, predicted CTCF binding sites, and regions with ESPERR (evolutionary and sequence pattern extraction through reduced representations) regulatory potential scores above 0.05), all as annotated in the UCSC Genome Browser [73,75], to remove the coding and regulatory parts of the genome, and to eliminate additional sequences evolving under functional constraints from the NCNR subgenome. Furthermore, all repeats identified by RepeatMasker [28], expect for mononucleotide microsatellites, were removed from the NCNR subgenome to exclude overlap with the AR subgenome. The AR subgenome consisted of all transposable elements that were inserted in the human genome before human-macaque divergence (excluding L1PA1-A7, L1HS, and *Alu*Y). The human genome was divided into 1-Mb windows, and coverage for both subgenomes was computed in each of the windows. Windows having less than 25% coverage of either NCNR sequences or ARs were discarded. Similarly, to perform analyses at finer scales, the human genome was divided into 0.5-Mb and 0.1-Mb windows and windows with less than 25% coverage of either NCNR sequences or ARs were discarded.

Alignments corresponding to the two subgenomes were fetched and pre-processed using tools from Galaxy (see Toolset section). Human-orangutan pair-wise alignments were fetched and processed for substitution rate and microsatellite mutability computations, after being filtered for quality using orangutan PHRED scores (with a minimum quality threshold of 20) and for synteny (only those alignments blocks that contained orangutan chromosomes syntenic to the human chromosomes were considered). Similarly, human-orangutan-macaque alignments were fetched and prepared for insertion and deletion rate computations. The proportion of the two subgenomes covered by these alignments is summarized in Table S8 in Additional file 1.

### Estimating mutation rates

Indels that occurred in the human lineage since its divergence from the orangutan lineage were obtained from the human-orangutan-macaque alignments, and insertions were distinguished from deletions using macaque as an outgroup (see detailed methods in [8]). Indels that overlapped with microsatellites were discarded to avoid scoring the same events twice. The insertion rate for each 1-Mb window was computed as the ratio of the number of insertions in all indel-containing quality filtered NCNR (or AR) alignment blocks in that window to the total number of nucleotides in all alignment blocks present in that window. Similarly, the deletion rate was computed as the ratio of the number of deletions to the total number of nucleotides. Nucleotide substitutions were identified from the NCNR (or AR) human-orangutan alignments using the Jukes' and Cantor's (JC69) model (see detailed methods in [10]). The nucleotide substitution rate for each window was then computed as the ratio of the total number of such substitutions in the window to the total number of nucleotides in the alignment blocks falling in the window. Orthologous human-orangutan mononucleotide microsatellites were identified using a modified version of Sputnik [76] that allows detection of mononucleotide microsatellites having at least nine repeats (based on microsatellite thresholds determined by previous studies [70,77]), separated from each other by at least 10 bp, and having the same repeat motif at orthologous locations in both species. They were then grouped into repeat number bins of size 4 (for example, 9 to 12, 13 to 16, and so on), and the mutability of each group was computed following the methods described in [78]. Following Kelkar *et al*. [9], only groups with 30 or more microsatellites were considered to ensure accuracy in the estimation of mutability. To eliminate the effect of repeat number on mutability, the mutability values were regressed on average repeat number of the bins and the residuals obtained were considered for further analysis. Only mononucleotide microsatellites were considered, since sufficient numbers of other microsatellites (repeats of di-, tri-, or tetranucleotide motifs) could not be obtained in windows of 1-Mb or smaller. Any mutation occurring in overlapping alignment blocks was discarded to avoid counting the same locus more than once, which would otherwise lead to an inflation of mutation rates. The workflow for estimating mutation rates is depicted in Figure 4.

For human-macaque comparisons, indel rates were computed from human-macaque-marmoset alignments preprocessed as described above, with marmoset as the outgroup, and substitution rates and microsatellite mutability were computed from pre-processed human-macaque pairwise alignments.

### Aggregating genomic landscape features

Genomic features such as GC content, number of CpG islands, male and female recombination rates, distance to telomere, number of LINEs, number of SINEs, SNP density and number of nuclear lamina binding sites were obtained from the UCSC Genome Browser per 1-Mb window of the human genome, based on hg18 annotations of the human genome in the UCSC Genome Browser. GC content was obtained from the gc5base program available from the UCSC Genome Browser, which computed the percentage of G and C nucleotides in 1-Mb windows. CpG islands were obtained from the cpgIslandExt table [79], and their counts were apportioned into 1-Mb windows using Galaxy tools. Male and female recombination rates in 1-Mb windows were obtained from the deCODE map rates [60] from the recombRate table. The coordinates of LINE and SINE elements were obtained from the RepeatMasker track and their respective counts in each 1-Mb window were computed using Galaxy tools. SNPs were obtained from the SNP129 track [80]. Nuclear lamina-associated sites were obtained from the 'NKI LaminB1' track [15] and were apportioned into 1-Mb windows after filtering out non-positive intensities and therefore retaining only those domains that are strongly bound to the lamina. Replication timing was calculated as the time spent by a sequence in the S phase of the cell [33]. Genomic coordinates of non-CpG methyl-cytosines were obtained from the datasets produced by Lister and colleagues [16]. Since the majority of the chromosomes between human and orangutan genomes have little or no chromosomal rearrangements [81], only the human telomere coordinates were considered to calculate distances to telomere. Telomere coordinates were obtained from the Gap track, and the distance from the middle of each window to its closest telomere was computed. Nucleosome-free regions predicted from MNase cleavage [17] were obtained, and their density in 1-Mb windows was computed using Galaxy tools. Coordinates of coding exons were obtained from the UCSC Genes track, and their coverage per window was computed using Galaxy tools. Similarly, we obtained coordinates of most conserved regions from the '28-way most conserved' track, removed coding exons from this list and obtained coverage per window using Galaxy tools.

At 0.5-Mb and 0.1-Mb scales, all features except recombination rate were computed as described above. Recombination rates computationally predicted from human genetic variation data [82] were used for 0.5-Mb and 0.1-Mb windows, as sex-specific rates are not available at these scales.

### Multivariate analysis

#### Normality and outliers

The resulting datasets consisting of aggregated genomic variables and mutation rates computed for the AR and NCNR subgenomes, separately, were each tested for conformity to multivariate normality, and subjected to multivariate outlier detection. As with simpler tools, multivariate techniques can be used in a purely descriptive manner. However, when tests of significance are required, and more generally if the data depart dramatically from multivariate normality, results may be misleading and difficult to interpret [31]. Our dataset was tested for conformity to multivariate normality based on a quantile-quantile (Q-Q) plot of ordered squared-robust Mahalanobis distances of the observations against the quantiles of a chi-squared distribution with degrees of freedom equal to the number of variables in the dataset. Mahalanobis distances give a measure of the distance of a particular observation from the mean vector of the sample, and take into account the covariance matrix - thereby quantifying both the shape and size of multivariate data [83]. If the observations follow multivariate normality, then these distances are known to have a chi-squared distribution with q degrees of freedom (where q is the number of variables in the dataset) [31]. The Q-Q plot for our dataset did not depart substantially from a straight line through the origin (data not shown), indicating conformity to multivariate normality.

Outliers may also substantially affect the results of multivariate techniques. We identified outliers as observations having large squared Mahalanobis distances based on a 90% quantile of the chi-squared distribution using the 'mvoutlier' package in R [84]. After removing outliers, we retained a total of 2,027 windows in ARs, and 1,953 windows in NCNR sequences, which were considered for all subsequent analyses.

Normality tests and outlier filtering were performed on windows at all genomic scales and all phylogenetic branches and the resulting statistics are summarized in Table S9 in Additional file 1.

#### Principal component analysis

PCA [31,34] extracts linear combinations of maximal variance in a given space of variables. The first principal component represents the linear combination whose variance in the data cloud is greatest amongst all possible linear combinations. The second principal component is constructed to be orthogonal to the first principal component, and to account for maximal variance after it, and so on. We performed PCA in the four-dimensional space of mutation rates using the *princomp *function from the R statistical package [85]. The principal components were extracted based on the correlation matrix (not the covariance matrix), and were therefore unaffected by units of measurement, scale and location of the different mutation rates. Of the four principal components, only the first two had eigenvalues (variances) ≥ 1 (the average eigenvalue of a correlation matrix), and they accounted for nearly 75% of the total variance. Therefore, following Kaiser's rule [29], we decided to consider only the first two principal components. The eigenvectors (loadings), which capture the correlations between principal components and original variables, were used to interpret the results of PCA.

#### Kernel principal component analysis

Gaussian kPCA [30] was performed using the R package 'kernlab' [86]. kPCA is a non-linear version of PCA, which employs a kernel function (K(x,x') = exp(-σ ||x - x'||^2^) in the Gaussian case) to calculate the inner products between data points in a high dimensional space F representing non-linearity, without actually performing the mapping to this space (this reduces computational burden). PCA is thus performed on F. The kernel function shown above is a general-purpose Gaussian radial basis function, which is normally used when no prior knowledge is available about the structure of the data. To determine if the signals obtained from kernel PCA are comparable with those from linear PCA, we obtained the scores of the observations (genomic windows) on the strongest kernel principal component and regressed them against the scores of the observations on the significant linear principal components. This allowed us to quantify how well the kernel scores of our genomic windows were recapitulated by their linear scores, that is, how comparable non-linear and linear signals were in the windows.

#### Canonical correlation analysis

CCA [31,34] extracts linearly correlated features from two sets of variables, both multidimensional in nature (that is, several Xs versus several Ys). It involves identifying pairs of maximally correlated canonical variates (CVs; u_i_,v_i_), where u_i _is a linear combination of the Xs, and v_i _a linear combination of the Ys. The first CV pair (u_1_,v_1_) has highest correlation R_1 _among all possible linear combinations of Xs and Ys. Similarly, the second CV pair (u_2_,v_2_) has the second largest correlation R_2_, with the constraint that u_2 _is orthogonal to u_1_, and v_2 _to v_1_, and so on. In all, s pairs (u_1_,v_1_), (u_2_,v_2_),...,(u_s_,v_s_) are extracted, such that R_1 _> R_2 _> ... > R_s_, where s is the smallest between the number of Xs and number of Ys.

We performed CCA with the aid of functions from the R package 'yacca' [87]. Four CVs were obtained, the statistical significance of which was assessed using an F test for canonical correlations, with Rao's approximation [34]. Loadings, which represent the correlations between the original variables and the respective CVs, were used to interpret the CVs in terms of the original X and Y variables.

#### Kernel canonical correlation analysis

kCCA [35] is a non-linear version of CCA, which uses a kernel function to provide maximally correlated non-linear features from the two sets of variables. We implemented it with the R 'kernlab' package employing again a Gaussian radial basis function as kernel.

For the strongest kernel canonical component, we used standardized predictor (or response) variable coefficients for each observation as an indicator of how strongly the observation is scored by the kernel component. These scores were then regressed against standardized predictor (or response) variable scores obtained from significant linear components to understand the extent to which non-linear and linear signals were compatible.

#### Comparison of linear and non-linear PCA (or CCA) scores

We regressed the 'strongest' non-linear signal (scores from kPCA or kCCA) onto significant linear signals (scores from PCA or CCA) to identify the extent to which the non-linear signal was being captured by the linear signals. Windows for which the standardized absolute residuals were greater than 2 were considered as drivers of non-linearity, that is, locations at which the non-linear signal was poorly recapitulated by the linear ones.

### Toolset in Galaxy

The following software tools are made available under the 'Regional variation', 'Multiple regression', and 'Multivariate analysis' tool sections of Galaxy.

#### Alignment data preprocessing

These are general-purpose tools, which can be used to process multiple genomic alignments of any species. We contributed tools to filter multiple alignments based on PHRED quality scores available for each sequenced genome at the UCSC Genome Browser [73,75], and to mask CpG or non-CpG sites in multiple alignments. For the first, quality scores for several species are locally cached in Galaxy, and the user is provided with options to select which species to mask, what quality cutoffs to use, how many positions surrounding low-quality bases to mask, and so on. For the second, the user can select an inclusive or restrictive definition of CpG sites [11], as well as the species on which to base the masking.

#### Computation of mutation rates

These tools allow the computation of nucleotide substitutions and microsatellite mutability from pair-wise alignments, and the computation of rates of insertions and deletions from three-way alignments. The mutations identified by these tools can be aggregated in genomic windows, and mutation rates per window can be calculated.

#### Aggregation of genomic features

Galaxy provides a direct connection to the UCSC Genome Browser [73,75], which houses genomic sequences and annotation data for numerous genomes. Genomic features retrieved into Galaxy from the UCSC table browser can be aggregated in windows of user-defined size by using the 'Make windows' and 'Feature coverage' tools in the 'Regional variation' section.

#### Statistical analyses

Tools for performing multiple regression, best subsets selection, and to compute RCVE (relative contribution to variability explained, a measure of the role of each predictor in explaining the total variability of a response) [8], are available in the 'Multiple regression' section. Besides providing summary regression output, these tools produce a number of diagnostic plots. Tools for performing linear and kernel PCA and CCA are available in the 'Multivariate analysis' section. These tools give the user several convenient options (for example, which variables to include, whether to scale the variables, what type of kernel to use) and produce summary output and graphics.

## Abbreviations

AR: ancestral repeats; bp: base pair; CCA: canonical correlation analysis; CV: canonical variate; indel, insertion and deletion; kCCA: kernel canonical correlation analysis; kPCA: kernel principal component analysis; LINE: long interspersed repetitive elements; Mb: megabase; NCNR: non-coding non-repetitive; PCA: principal component analysis; SINE: short interspersed repetitive element; SNP: single nucleotide polymorphism.

## Authors' contributions

All authors conceived and designed the analysis framework. GA implemented and performed the analyses. All authors participated in interpretation of results. All authors read and approved the final manuscript.

## Supplementary Material

Additional file 1 **Figures and tables depicting PCA and CCA results along the human-macaque and mouse-rat branches at 1-Mb, 0.5-Mb, and 0.1-Mb scales, and along the human-orangutan branch at 0.5-Mb and 0.1-Mb scales**. Click here for file

## References

[B1] Lercher MJ Williams EJ Hurst LD Local similarity in evolutionary rates extends over whole chromosomes in human-rodent and mouse-rat comparisons: implications for understanding the mechanistic basis of the male mutation bias. Mol Biol Evol 2001 18 2032 2039 11606699 10.1093/oxfordjournals.molbev.a003744

[B2] Hardison RC Roskin KM Yang S Diekhans M Kent WJ Weber R Elnitski L Li J O'Connor M Kolbe D Schwartz S Furey TS Whelan S Goldman N Smit A Miller W Chiaromonte F Haussler D Covariation in frequencies of substitution, deletion, transposition, and recombination during eutherian evolution. Genome Res 2003 13 13 26 10.1101/gr.844103 12529302 PMC430971

[B3] Ellegren H Microsatellites: simple sequences with complex evolution. Nat Rev Genet 2004 5 435 445 10.1038/nrg1348 15153996

[B4] Makova KD Yang S Chiaromonte F Insertions and deletions are male biased too: a whole-genome analysis in rodents. Genome Res 2004 14 567 573 10.1101/gr.1971104 15059997 PMC383300

[B5] Lunter G Ponting CP Hein J Genome-wide identification of human functional DNA using a neutral indel model. PLoS Comput Biol 2006 2 e5 10.1371/journal.pcbi.0020005 16410828 PMC1326222

[B6] Hellmann I Prufer K Ji H Zody MC Paabo S Ptak SE Why do human diversity levels vary at a megabase scale? Genome Res 2005 15 1222 1231 10.1101/gr.3461105 16140990 PMC1199536

[B7] Webster MT Axelsson E Ellegren H Strong regional biases in nucleotide substitution in the chicken genome. Mol Biol Evol 2006 23 1203 1216 10.1093/molbev/msk008 16551647

[B8] Kvikstad EM Tyekucheva S Chiaromonte F Makova KD A macaque's-eye view of human insertions and deletions: differences in mechanisms. PLoS Comput Biol 2007 3 1772 1782 10.1371/journal.pcbi.0030176 17941704 PMC1976337

[B9] Kelkar YD Tyekucheva S Chiaromonte F Makova KD The genome-wide determinants of human and chimpanzee microsatellite evolution. Genome Res 2008 18 30 38 10.1101/gr.7113408 18032720 PMC2134767

[B10] Tyekucheva S Makova KD Karro JE Hardison RC Miller W Chiaromonte F Human-macaque comparisons illuminate variation in neutral substitution rates. Genome Biol 2008 9 R76 10.1186/gb-2008-9-4-r76 18447906 PMC2643947

[B11] Taylor J Tyekucheva S Zody M Chiaromonte F Makova KD Strong and weak male mutation bias at different sites in the primate genomes: insights from the human-chimpanzee comparison. Mol Biol Evol 2006 23 565 573 10.1093/molbev/msj060 16280537

[B12] Li WH Yi S Makova K Male-driven evolution. Curr Opin Genet Dev 2002 12 650 656 10.1016/S0959-437X(02)00354-4 12433577

[B13] Ellegren H Characteristics, causes and evolutionary consequences of male-biased mutation. Proc Biol Sci 2007 274 1 10 10.1098/rspb.2006.3720 17134994 PMC1679872

[B14] Linardopoulou EV Williams EM Fan Y Friedman C Young JM Trask BJ Human subtelomeres are hot spots of interchromosomal recombination and segmental duplication. Nature 2005 437 94 100 10.1038/nature04029 16136133 PMC1368961

[B15] Guelen L Pagie L Brasset E Meuleman W Faza MB Talhout W Eussen BH de Klein A Wessels L de Laat W van Steensel B Domain organization of human chromosomes revealed by mapping of nuclear lamina interactions. Nature 2008 453 948 951 10.1038/nature06947 18463634

[B16] Lister R Pelizzola M Dowen RH Hawkins RD Hon G Tonti-Filippini J Nery JR Lee L Ye Z Ngo QM Edsall L Antosiewicz-Bourget J Stewart R Ruotti V Millar AH Thomson JA Ren B Ecker JR Human DNA methylomes at base resolution show widespread epigenomic differences. Nature 2009 462 315 322 10.1038/nature08514 19829295 PMC2857523

[B17] Ozsolak F Song JS Liu XS Fisher DE High-throughput mapping of the chromatin structure of human promoters. Nat Biotechnol 2007 25 244 248 10.1038/nbt1279 17220878

[B18] Chiaromonte F Yang S Elnitski L Yap VB Miller W Hardison RC Association between divergence and interspersed repeats in mammalian noncoding genomic DNA. Proc Natl Acad Sci USA 2001 98 14503 14508 10.1073/pnas.251423898 11717405 PMC64711

[B19] Tian D Wang Q Zhang P Araki H Yang S Kreitman M Nagylaki T Hudson R Bergelson J Chen JQ Single-nucleotide mutation rate increases close to insertions/deletions in eukaryotes. Nature 2008 455 105 108 10.1038/nature07175 18641631

[B20] Pearson CE Nichol Edamura K Cleary JD Repeat instability: mechanisms of dynamic mutations. Nat Rev Genet 2005 6 729 742 10.1038/nrg1689 16205713

[B21] Yang S Smit AF Schwartz S Chiaromonte F Roskin KM Haussler D Miller W Hardison RC Patterns of insertions and their covariation with substitutions in the rat, mouse, and human genomes. Genome Res 2004 14 517 527 10.1101/gr.1984404 15059992 PMC383295

[B22] Rolfsmeier ML Lahue RS Stabilizing effects of interruptions on trinucleotide repeat expansions in *Saccharomyces cerevisiae*. Mol Cell Biol 2000 20 173 180 10.1128/MCB.20.1.173-180.2000 10594019 PMC85072

[B23] Taylor J Schenck I Blankenberg D Nekrutenko A Using galaxy to perform large-scale interactive data analyses. Curr Protoc Bioinformatics 2007 10 Unit 10.5 18428782 10.1002/0471250953.bi1005s19 PMC3418382

[B24] Makova KD Li WH Strong male-driven evolution of DNA sequences in humans and apes. Nature 2002 416 624 626 10.1038/416624a 11948348

[B25] Gaffney DJ Keightley PD The scale of mutational variation in the murid genome. Genome Res 2005 15 1086 1094 10.1101/gr.3895005 16024822 PMC1182221

[B26] Kvikstad EM Makova KD The (r)evolution of SINE vs. LINE distributions in primate genomes: Sex chromosomes are important. Genome Res 2010 20 600 613 10.1101/gr.099044.109 20219940 PMC2860162

[B27] Webster MT Smith NGC Lercher MJ Ellegren H Gene expression, synteny, and local similarity in human noncoding mutation rates. Mol Biol Evol 2004 21 1820 1830 10.1093/molbev/msh181 15175414

[B28] RepeatMasker http://www.repeatmasker.org/

[B29] Kaiser HF The varimax criterion for analytic rotation in factor analysis. Psychometrika 1958 23 187 200 10.1007/BF02289233

[B30] Scholkopf B Smola A Muller KR Nonlinear component analysis as a kernel eigenvalue problem. Neural Computation 1998 10 1299 1319 10.1162/089976698300017467

[B31] Everitt BS An R and S-Plus Companion to Multivariate Analysis 2005 London: Springer

[B32] Deschavanne P Filipski J Correlation of GC content with replication timing and repair mechanisms in weakly expressed *E. coli *genes. Nucleic Acids Res 1995 23 1350 1353 10.1093/nar/23.8.1350 7753625 PMC306860

[B33] Woodfine K Fiegler H Beare DM Collins JE McCann OT Young BD Debernardi S Mott R Dunham I Carter NP Replication timing of the human genome. Hum Mol Genet 2004 13 191 202 10.1093/hmg/ddh016 14645202

[B34] Mardia KV Kent JT Bibby JM Multivariate Analysis 1979 London: Academic Press

[B35] Kuss M Graepel T Technical Report No. 108: The Geometry of Kernel Canonical Correlation Analysis 2003 Tübingen, Germany: Max Planck Institute for Biological Cybernetics http://www.kernel-machines.org/papers/upload_22685_TR-108.pdf

[B36] Mouse Genome Sequencing Consortium Initial sequencing and comparative analysis of the mouse genome. Nature 2002 420 520 562 10.1038/nature01262 12466850

[B37] Wetterbom A Sevov M Cavelier L Bergstrom TF Comparative genomic analysis of human and chimpanzee indicates a key role for indels in primate evolution. J Mol Evol 2006 63 682 690 10.1007/s00239-006-0045-7 17075697

[B38] Walsh CP Bestor TH Cytosine methylation and mammalian development. Genes Dev 1999 13 26 34 10.1101/gad.13.1.26 9887097 PMC316374

[B39] Cross SH Bird AP CpG islands and genes. Curr Opin Genet Dev 1995 5 309 314 10.1016/0959-437X(95)80044-1 7549424

[B40] The Chimpanzee Sequencing and Analysis Consortium Initial sequence of the chimpanzee genome and comparison with the human genome. Nature 2005 437 69 87 10.1038/nature04072 16136131

[B41] Saccone S Federico C Bernardi G Localization of the gene-richest and the gene-poorest isochores in the interphase nuclei of mammals and birds. Gene 2002 300 169 178 10.1016/S0378-1119(02)01038-7 12468098

[B42] Di Filippo M Bernardi G Mapping DNase-I hypersensitive sites on human isochores. Gene 2008 419 62 65 10.1016/j.gene.2008.02.012 18436395

[B43] Costantini M Bernardi G Mapping insertions, deletions and SNPs on Venter's chromosomes. PLoS One 2009 4 e5972 10.1371/journal.pone.0005972 19543403 PMC2696090

[B44] Weiner AM SINEs and LINEs: the art of biting the hand that feeds you. Curr Opin Cell Biol 2002 14 343 350 10.1016/S0955-0674(02)00338-1 12067657

[B45] Boulikas T Evolutionary consequences of nonrandom damage and repair of chromatin domains. J Mol Evol 1992 35 156 180 10.1007/BF00183227 1501255

[B46] Bohr VA Phillips DH Hanawalt PC Heterogeneous DNA damage and repair in the mammalian genome. Cancer Res 1987 47 6426 6436 3315187

[B47] Filipski J Correlation between molecular clock ticking, codon usage fidelity of DNA repair, chromosome banding and chromatin compactness in germline cells. FEBS Lett 1987 217 184 186 10.1016/0014-5793(87)80660-9 3595849

[B48] Duret L Arndt PF The impact of recombination on nucleotide substitutions in the human genome. PLoS Genet 2008 4 e1000071 10.1371/journal.pgen.1000071 18464896 PMC2346554

[B49] Duret L Galtier N Biased gene conversion and the evolution of mammalian genomic landscapes. Annu Rev Genomics Hum Genet 2009 10 285 311 10.1146/annurev-genom-082908-150001 19630562

[B50] Bill CA Duran WA Miselis NR Nickoloff JA Efficient repair of all types of single-base mismatches in recombination intermediates in Chinese hamster ovary cells: Competition between long-patch and G-T glycosylase-mediated repair of G-T mismatches. Genetics 1998 149 1935 1943 9691048 10.1093/genetics/149.4.1935 PMC1460289

[B51] Brown TC Jiricny J Different base/base mispairs are corrected with different efficiencies and specificities in monkey kidney cells. Cell 1988 54 705 711 10.1016/S0092-8674(88)80015-1 2842064

[B52] Zhu YY Strassmann JJE Queller DDC Insertions, substitutions, and the origin of microsatellites. Genet Res 2000 76 227 236 10.1017/S001667230000478X 11204970

[B53] International Chicken Genome Sequencing Consortium Sequence and comparative analysis of the chicken genome provide unique perspectives on vertebrate evolution. Nature 2004 432 695 716 10.1038/nature03154 15592404

[B54] Ross MT Grafham DV Coffey AJ Scherer S McLay K Muzny D Platzer M Howell GR Burrows C Bird CP Frankish A Lovell FL Howe KL Ashurst JL Fulton RS Sudbrak R Wen G Jones MC Hurles ME Andrews TD Scott CE Searle S Ramser J Whittaker A Deadman R Carter NP Hunt SE Chen R Cree A Gunaratne P The DNA sequence of the human X chromosome. Nature 2005 434 325 337 10.1038/nature03440 15772651 PMC2665286

[B55] Skaletsky H Kuroda-Kawaguchi T Minx PJ Cordum HS Hillier L Brown LG Repping S Pyntikova T Ali J Bieri T Chinwalla A Delehaunty A Delehaunty K Du H Fewell G Fulton L Fulton R Graves T Hou SF Latrielle P Leonard S Mardis E Maupin R McPherson J Miner T Nash W Nguyen C Ozersky P Pepin K Rock S The male-specific region of the human Y chromosome is a mosaic of discrete sequence classes. Nature 2003 423 825 837 10.1038/nature01722 12815422

[B56] Abrusan G Krambeck HJ Junier T Giordano J Warburton PE Biased distributions and decay of long interspersed nuclear elements in the chicken genome. Genetics 2008 178 573 581 10.1534/genetics.106.061861 17947446 PMC2206104

[B57] Boissinot S Entezam A Furano AV Selection against deleterious LINE-1-containing loci in the human lineage. Mol Biol Evol 2001 18 926 935 11371580 10.1093/oxfordjournals.molbev.a003893

[B58] Jurka J Kohany O Pavlicek A Kapitonov VV Jurka MV Duplication, coclustering, and selection of human Alu retrotransposons. Proc Natl Acad Sci USA 2004 101 1268 1272 10.1073/pnas.0308084100 14736919 PMC337042

[B59] Jensen-Seaman MI Furey TS Payseur BA Lu Y Roskin KM Chen CF Thomas MA Haussler D Jacob HJ Comparative recombination rates in the rat, mouse, and human genomes. Genome Res 2004 14 528 538 10.1101/gr.1970304 15059993 PMC383296

[B60] Kong A Gudbjartsson DF Sainz J Jonsdottir GM Gudjonsson SA Richardsson B Sigurdardottir S Barnard J Hallbeck B Masson G Shlien A Palsson ST Frigge ML Thorgeirsson TE Gulcher JR Stefansson K A high-resolution recombination map of the human genome. Nat Genet 2002 31 241 247 12053178 10.1038/ng917

[B61] Yu A Zhao C Fan Y Jang W Mungal AJ Deloukas P Olsen A Doggett NA Ghebranious N Broman KW Weber JL Comparison of human genetic and sequence-based physical maps. Nature 2000 409 951 953 10.1038/35057185 11237020

[B62] Rudd MK Friedman C Parghi SS Linardopoulou EV Hsu L Trask BJ Elevated rates of sister chromatid exchange at chromosome ends. PLoS Genet 2007 3 e32 10.1371/journal.pgen.0030032 17319749 PMC1802831

[B63] Eory L Halligan DL Keightley PD Distributions of selectively constrained sites and deleterious mutation rates in the hominid and murid genomes. Mol Biol Evol 2010 27 177 192 10.1093/molbev/msp219 19759235

[B64] Lieberman-Aiden E van Berkum NL Williams L Imakaev M Ragoczy T Telling A Amit I Lajoie BR Sabo PJ Dorschner MO Sandstrom R Bernstein B Bender MA Groudine M Gnirke A Stamatoyannopoulos J Mirny LA Lander ES Dekker J Comprehensive mapping of long-range interactions reveals folding principles of the human genome. Science 2009 326 289 293 10.1126/science.1181369 19815776 PMC2858594

[B65] Amos W Heterozygosity and mutation rate: evidence for an interaction and its implications. Bioessays 2010 32 82 90 10.1002/bies.200900108 19967709

[B66] Amos W Even small SNP clusters are non-randomly distributed: is this evidence of mutational non-independence? Proc Biol Sci 2010 277 1443 1449 10.1098/rspb.2009.1757 20071383 20071383 PMC2871933

[B67] Amos W Flint J Xu X Heterozygosity increases microsatellite mutation rate, linking it to demographic history. BMC Genet 2008 9 72 10.1186/1471-2156-9-72 19014581 PMC2615044

[B68] Stamatoyannopoulos JA Adzhubei I Thurman RE Kryukov GV Mirkin SM Sunyaev SR Human mutation rate associated with DNA replication timing. Nat Genet 2009 41 393 395 10.1038/ng.363 19287383 PMC2914101

[B69] Huang SW Friedman R Yu N Yu A Li WH How strong is the mutagenicity of recombination in mammals? Mol Biol Evol 2005 22 426 431 10.1093/molbev/msi025 15496551

[B70] Kelkar YD Strubczewski N Hile SE Chiaromonte F Eckert KA Makova KD What is a microsatellite: a computational and experimental definition based upon repeat mutational behavior at A/T and GT/AC repeats. Genome Biol Evol 2010 2 620 635 10.1093/gbe/evq046 20668018 PMC2940325

[B71] Siepel A Bejerano G Pedersen JS Hinrichs AS Hou MM Rosenbloom K Clawson H Spieth J Hillier LW Richards S Weinstock GM Wilson RK Gibbs RA Kent WJ Miller W Haussler D Evolutionarily conserved elements in vertebrate, insect, worm, and yeast genomes. Genome Res 2005 15 1034 1050 10.1101/gr.3715005 16024819 PMC1182216

[B72] Taylor J Tyekucheva S King DC Hardison RC Miller W Chiaromonte F ESPERR: Learning strong and weak signals in genomic sequence alignments to identify functional elements. Genome Res 2006 16 1596 1604 10.1101/gr.4537706 17053093 PMC1665643

[B73] Karolchik D Kuhn RM Baertsch R Barber GP Clawson H Diekhans M Giardine B Harte RA Hinrichs AS Hsu F Kober KM Miller W Pedersen JS Pohl A Raney BJ Rhead B Rosenbloom KR Smith KE Stanke M Thakkapallayil A Trumbower H Wang T Zweig AS Haussler D Kent WJ The UCSC Genome Browser Database: 2008 update. Nucleic Acids Res 2008 36 D773 779 10.1093/nar/gkm966 18086701 PMC2238835

[B74] Hsu F Kent WJ Clawson H Kuhn RM Diekhans M Haussler D The UCSC Known Genes. Bioinformatics 2006 22 1036 1046 10.1093/bioinformatics/btl048 16500937

[B75] Kent WJ Sugnet CW Furey TS Roskin KM Pringle TH Zahler AM Haussler D The human genome browser at UCSC. Genome Res 2002 12 996 1006 12045153 10.1101/gr.229102 PMC186604

[B76] Sputnik http://espressosoftware.com/sputnik/index.html

[B77] Lai Y Sun F The relationship between microsatellite slippage mutation rate and the number of repeat units. Mol Biol Evol 2003 20 2123 2131 10.1093/molbev/msg228 12949124

[B78] Webster MT Smith NG Ellegren H Microsatellite evolution inferred from human-chimpanzee genomic sequence alignments. Proc Natl Acad Sci USA 2002 99 8748 8753 10.1073/pnas.122067599 12070344 PMC124370

[B79] Gardinergarden M Frommer M Cpg Islands in Vertebrate Genomes. J Mol Biol 1987 196 261 282 10.1016/0022-2836(87)90689-9 3656447

[B80] Sherry ST Ward MH Kholodov M Baker J Phan L Smigielski EM Sirotkin K dbSNP: the NCBI database of genetic variation. Nucleic Acids Res 2001 29 308 311 10.1093/nar/29.1.308 11125122 PMC29783

[B81] Muller S Wienberg J "Bar-coding" primate chromosomes: molecular cytogenetic screening for the ancestral hominoid karyotype. Hum Genet 2001 109 85 94 10.1007/s004390100535 11479739

[B82] Myers S Bottolo L Freeman C McVean G Donnelly P A fine-scale map of recombination rates and hotspots across the human genome. Science 2005 310 321 324 10.1126/science.1117196 16224025

[B83] Filzmoser P Garrett RG Clemens R Multivariate outlier detection in exploration geochemistry. Computers Geosci 2005 31 579 587 10.1016/j.cageo.2004.11.013

[B84] Gschwandtner M Filzmoser P mvoutlier: Multivariate outlier detection based on robust methods. R package version 1.4. 2009 http://medipe.psu.ac.th/cran-r/web/packages/mvoutlier/index.html

[B85] R Development Core Team R: A language and environment for statistical computing. 2009 Vienna, Austria: R Foundation for Statistical Computing

[B86] Karatzoglou A Smola A Hornik K Zeileis A kernlab - an S4 package for kernel methods in R. J Stat Software 2004 11 1 20 http://www.jstatsoft.org/v11/i09/paper

[B87] Butts CT yacca: Yet Another Canonical Correlation Analysis Package. R package version 1.1. 2009 http://cran.r-project.org/web/packages/yacca/index.html

