# Supplementary material for: A genome-wide view of mutation rate co-variation using multivariate analyses
Source: Genome Biol. 2011 Mar 22;12(3):R27. doi: 10.1186/gb-2011-12-3-r27 (PMC3129677; doi:10.1186/gb-2011-12-3-r27)
Supplement: Additional file 1 — Figures and tables depicting PCA and CCA results along the human-macaque and mouse-rat branches at 1-Mb, 0.5-Mb, and 0.1-Mb scales, and along the human-orangutan branch at 0.5-Mb and 0.1-Mb scales. [file gb-2011-12-3-r27-S1.PDF]

SUPPLEMENTARY FIGURE LEGENDS

Figure S1. Scatterplot of number of orthologous microsatellites (Y) against substitution rate (X) measured in 1-Mb windows. The regression line with intercept = 97.95 (*p*-value < 2e-16) and slope = -1475.66 (*p*-value < 2e-16) is plotted in red.

Figure S2. Scatterplots of each rotated mutation rate from the first component of kPCA (Y) against the original rate variable (X) for the AR sub-genome. Black dots represent observations (i.e. 1-Mb windows). Lowess (locally weighted scatterplot smoothing) fits to the data are plotted in red. INS, DEL, SUB, and MS stand for insertion rate, deletion rate, substitution rate, and mononucleotide microsatellite mutability, respectively. INS, DEL and SUB all appear to follow a sigmoid curve (the shape here is imposed by the choice of kernel), whereas MS does not – implying that the former three have strong loadings for this principal component.

Figure S3. Same as Fig. S2, but for the NCNR sub-genome.

Figure S4. Scores from the first non-linear component (Y) are plotted against the fitted values from the regression of these scores on significant linear components (X), for the AR (A) and NCNR (B) sub-genomes. Kp1, Ky1 and Kx1 represent the dominant non-linear signals obtained from kPCA, kCCA in the response space and kCCA in the predictor space, respectively. The number of linear and non-linear (i.e. outlying) loci (i.e. windows) are indicated in the figure legend. See *Tables S4-S5* for summary statistics.

Figure S5. Genome-wide locations of linear and non-linear windows for the AR sub-genome. Black circles denote linear windows. Green and blue circles denote windows displaying non-linear signals in mutation rates when considering PCA (A) and CCA in the response space (B) respectively. Red circles denote windows displaying non-linear signals in genomic features when considering CCA in the predictor space (C). Yellow triangles represent the location of the centromere along the chromosomes.

Figure S6. Same as Fig. S5, but for the NCNR sub-genome.

Figure S7. Biplots of the first three significant components (Component 1 vs. Component 2 (A), Component 2 vs. Component 3 (B), Component 1 vs. Component 3 (C)) obtained applying PCA to our genomic landscape variables in the AR and NCNR sub-genomes. GC – GC content, CpG – number of CpG islands, nCGm – number of methyl-cytosines in non-CpG context, LINE – number of LINE elements, SINE – number of SINE elements, NLp – number of nuclear lamina associated regions, Telo – distance to the telomere, fRec and mRec – female and male recombination rates respectively, SNPd – SNP density, RepT – replication time, nucFree – density of nucleosome-free regions. See *Tables S4-S5* for summary statistics.

Figure S8. Scatterplots of the first kCCA scores in response (mutation rates; Y) and predictor (genomic features; X) spaces, each against the closest (most strongly correlated) corresponding CCA score, for the AR (A) and NCNR (B) sub-genomes. Lowess (locally weighted scatterplot smoothing) fits to the data are plotted in red. Genomic features (X) show more non-linearity (curvature) in comparison to mutation rates (Y).

Figure S9. Biplots of the first two PCA components for our four mutation rates, as obtained from the NCNR sub-genome along the human-macaque (A) and mouse-rat (B) comparisons for 1-Mb windows. Black dots represent projected observations (i.e. projected windows). The vectors labeled INS, DEL, SUB, and MS depict loadings for insertion rate, deletion rate, substitution rate, and mononucleotide microsatellite mutability, respectively.

Figure S10. Biplots of the first two PCA components for our four mutation rates, as obtained from the AR and NCNR sub-genomes along human-orangutan comparison (A) and from the NCNR sub-genome for human-macaque (B) and mouse-rat (C) comparisons for 0.5-Mb windows. Black dots represent projected observations (i.e. projected windows). The vectors labeled INS, DEL, SUB, and MS depict loadings for insertion rate, deletion rate, substitution rate, and mononucleotide microsatellite mutability, respectively.

Figure S11. Biplots of the first two PCA components for our four mutation rates, as obtained from the AR and NCNR sub-genomes along human-orangutan comparison (A)

and from the NCNR sub-genome for human-macaque (B) and mouse-rat (C) comparisons for 0.1-Mb windows. Black dots represent projected observations (i.e. projected windows). The vectors labeled INS, DEL, SUB, and MS depict loadings for insertion rate, deletion rate, substitution rate, and mononucleotide microsatellite mutability, respectively.

Figure S12. Helioplots for CCA performed on the NCNR sub-genome along the human-macaque comparison for 1-Mb windows. The labels on the plots are as follows: CV – canonical variate, GC – GC content, CpG – number of CpG islands, nCGm – number of methyl-cytosines in non-CpG context, LINE – number of LINE elements, SINE – number of SINE elements, NLp – number of nuclear lamina associated regions, Telo – distance to the telomere, fRec and mRec – female and male recombination rates respectively, SNPd – SNP density, RepT – replication time, nucFree – density of nucleosome-free regions, cExon – coverage by coding exons, mostCons – coverage by most conserved elements. Red bars indicate positive loading, and blue bars negative loading.

Figure S13. Helioplots for CCA performed on the AR (A) and NCNR (B) sub-genomes along the human-orangutan comparison for 0.5-Mb windows. The labels on the plots are as follows: CV – canonical variate, GC – GC content, CpG – number of CpG islands, nCGm – number of methyl-cytosines in non-CpG context, LINE – number of LINE elements, SINE – number of SINE elements, NLp – number of nuclear lamina associated regions, Telo – distance to the telomere, rec – recombination rate, SNPd – SNP density, RepT – replication time, nucFree – density of nucleosome-free regions, cExon – coverage by coding exons, mostCons – coverage by most conserved elements. Red bars indicate positive loading, and blue bars negative loading.

Figure S14. Same as Fig. S13 but for the NCNR sub-genome along human-macaque comparison.

Figure S15. Helioplots for CCA performed on the AR (A) and NCNR (B) sub-genomes along the human-orangutan comparison for 0.1-Mb windows. The labels on the plots are as follows: CV – canonical variate, GC – GC content, CpG – number of CpG islands, nCGm – number of methyl-cytosines in non-CpG context, LINE – number of LINE elements, SINE – number of SINE elements, NLp – number of nuclear lamina

associated regions, Telo – distance to the telomere, rec – recombination rate, SNPd – SNP density, RepT – replication time, nucFree – density of nucleosome-free regions, cExon – coverage by coding exons, mostCons – coverage by most conserved elements. Red bars indicate positive loading, and blue bars negative loading.

Figure S16. Same as Fig. S15 but for the NCNR sub-genome along human-macaque comparison.

Figure S17. Helioplots for CCA performed on the NCNR sub-genome along the mouse-rat comparison for 1-Mb (A), 0.5-Mb (B) and 0.1-Mb (C) windows. The labels on the plots are as follows: CV – canonical variate, GC – GC content, CpG – number of CpG islands, LINE – number of LINE elements, SINE – number of SINE elements, Telo – distance to the telomere, fRec and mRec – female and male recombination rates respectively, SNPd – SNP density, cExon – coverage by coding exons, mostCons – coverage by most conserved elements. Red bars indicate positive loading, and blue bars negative loading.

Figure S1.

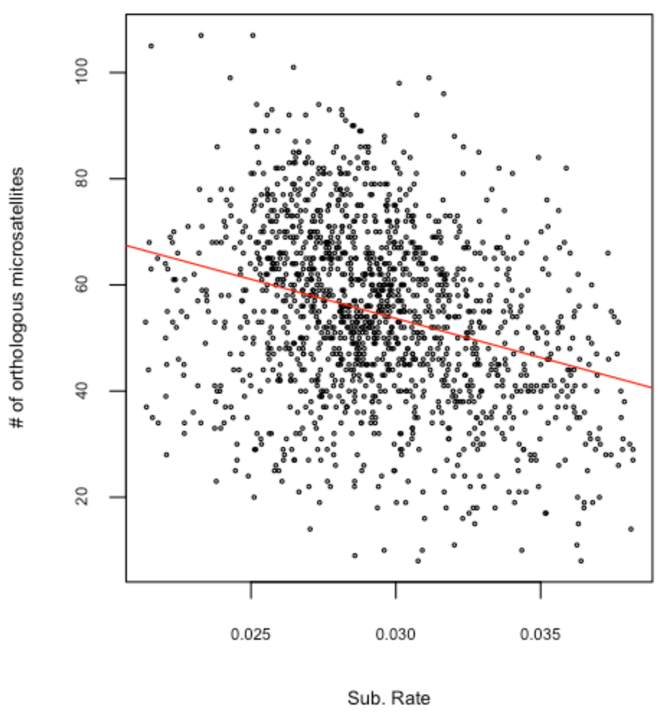

100
80
60
40
20
# of orthologous microsatellites
0.025
0.030
0.035
Sub. Rate

Figure S2.

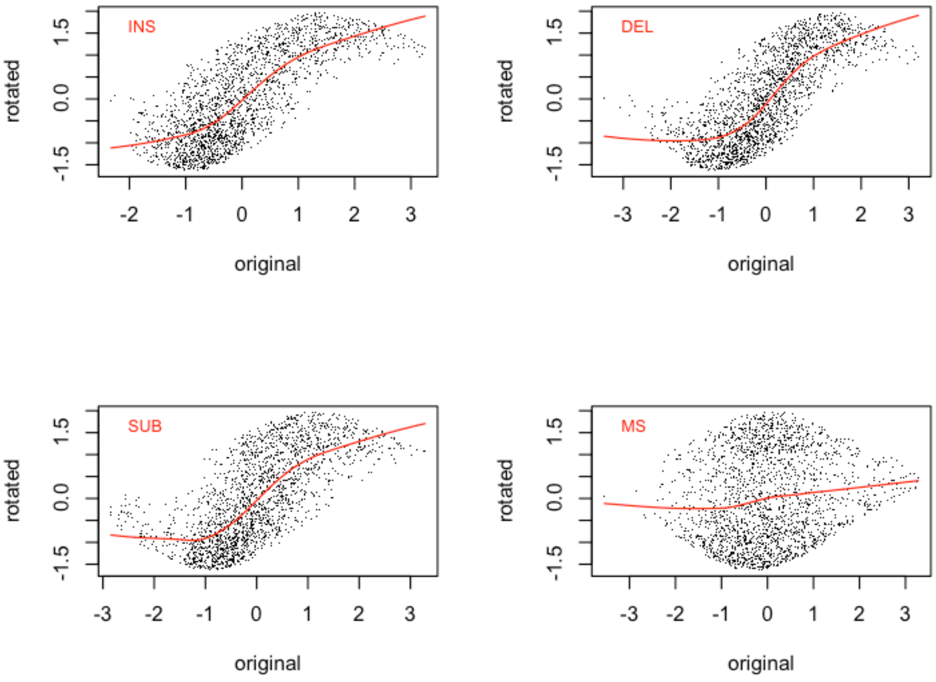

Figure S3.

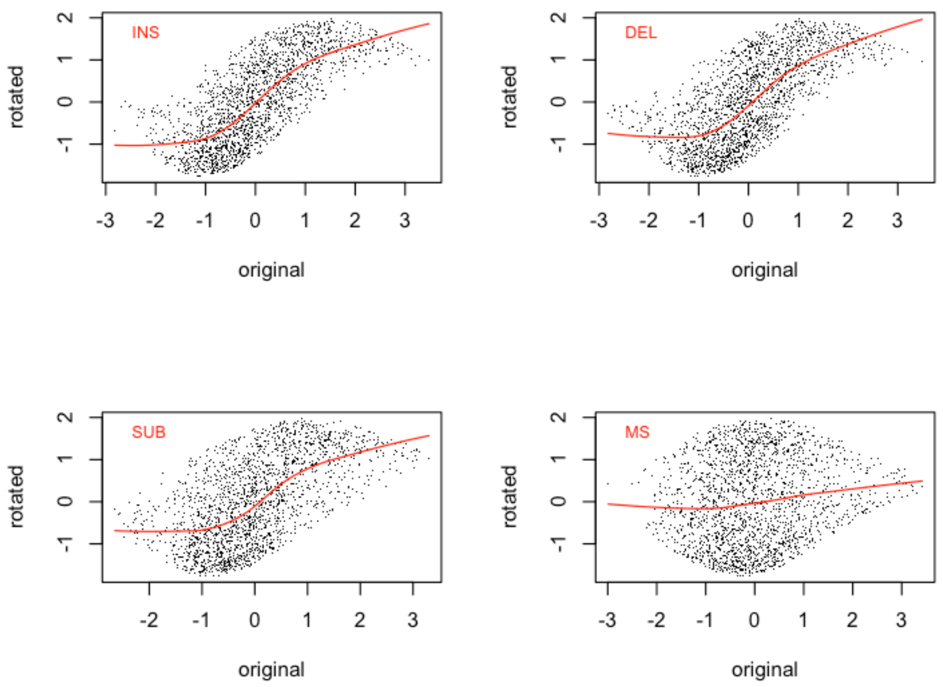

Figure S4.

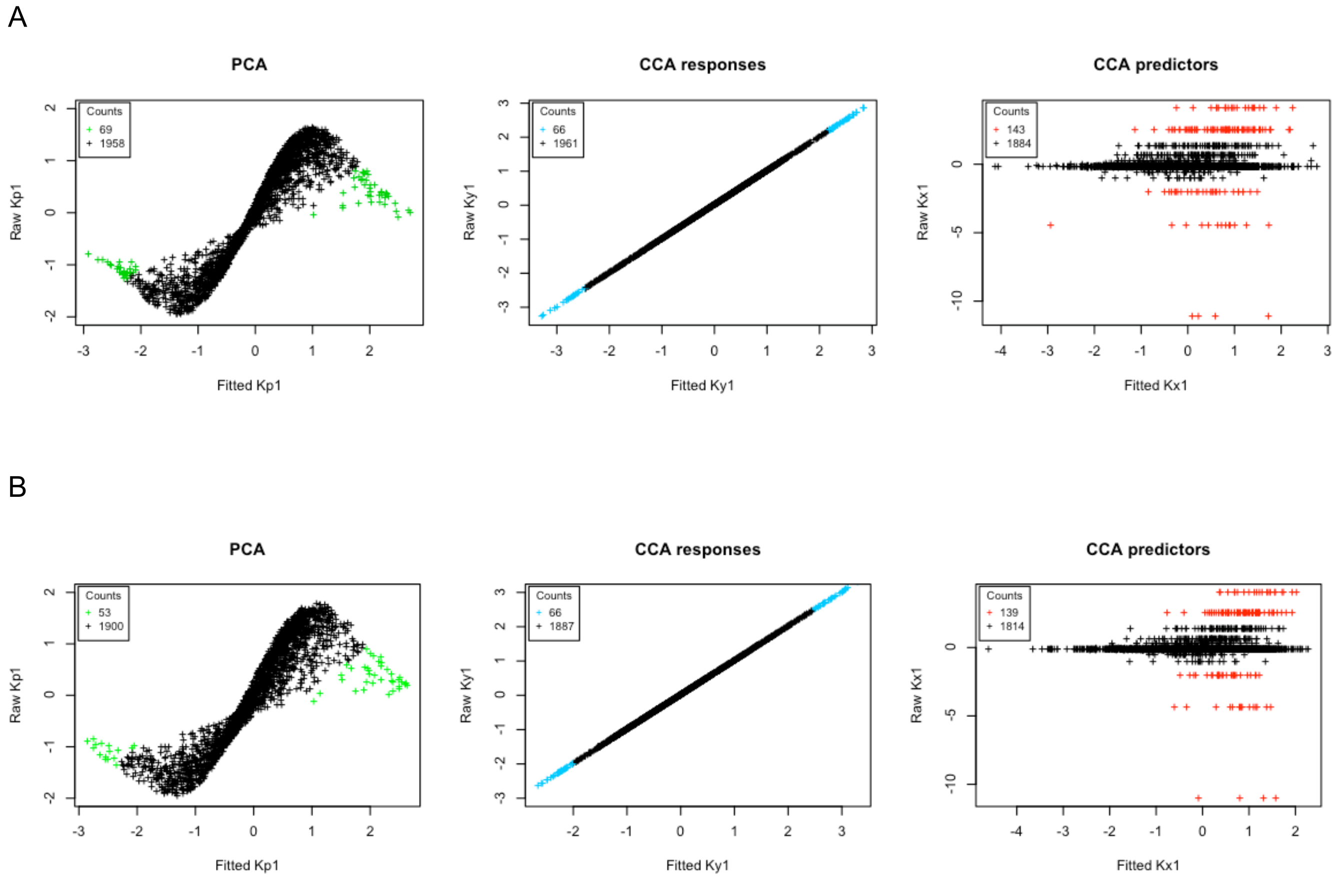
A
PCA
Counts
69
1958
Raw Kp1
Fitted Kp1
CCA responses
Counts
66
1961
Raw Ky1
Fitted Ky1
CCA predictors
Counts
143
1884
Raw Kx1
Fitted Kx1
B
PCA
Counts
53
1900
Raw Kp1
Fitted Kp1
CCA responses
Counts
66
1887
Raw Ky1
Fitted Ky1
CCA predictors
Counts
139
1814
Raw Kx1
Fitted Kx1

Figure S5.

A

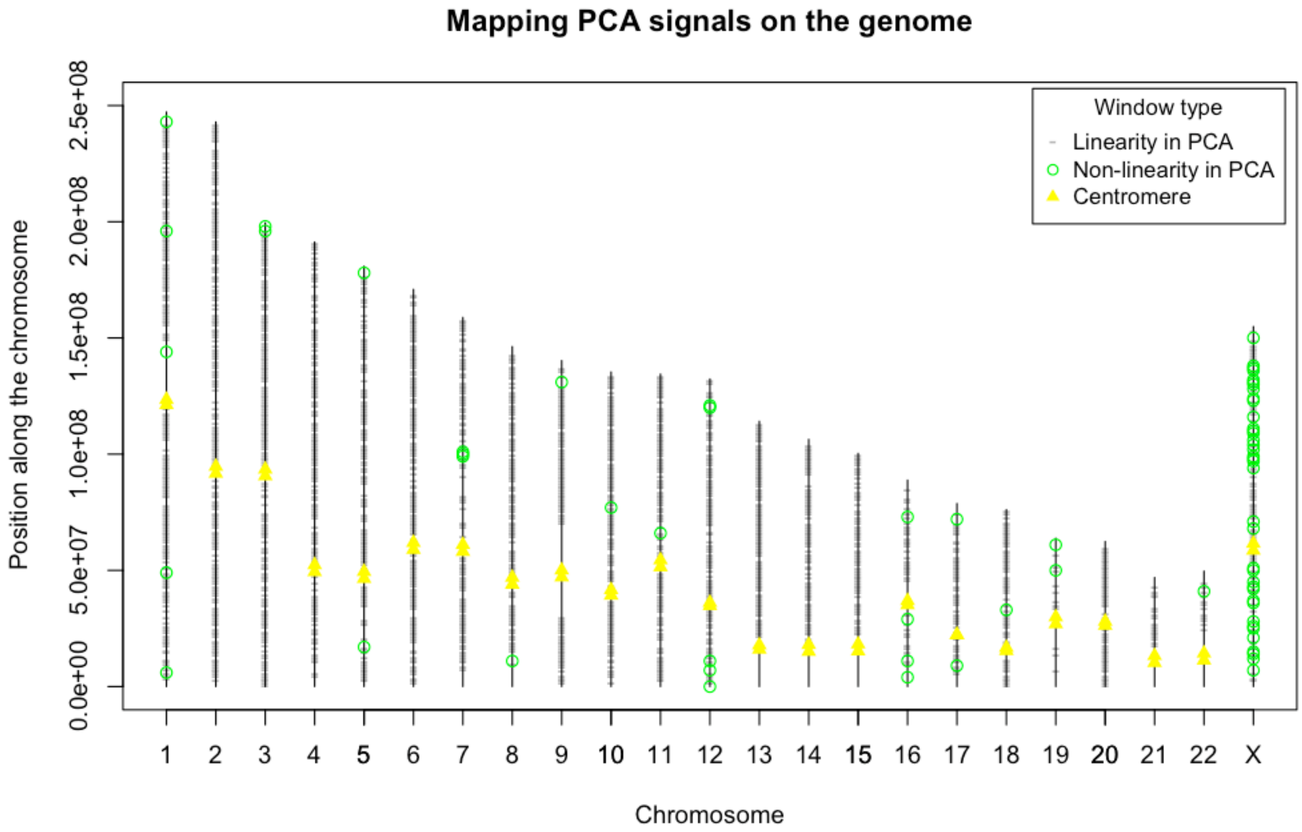

Mapping PCA signals on the genome
Window type
Linearity in PCA
Non-linearity in PCA
Centromere
Position along the chromosome
0.0e+00
5.0e+07
1.0e+08
1.5e+08
2.0e+08
2.5e+08
1
2
3
4
5
6
7
8
9
10
11
12
13
14
15
16
17
18
19
20
21
22
X
Chromosome

B

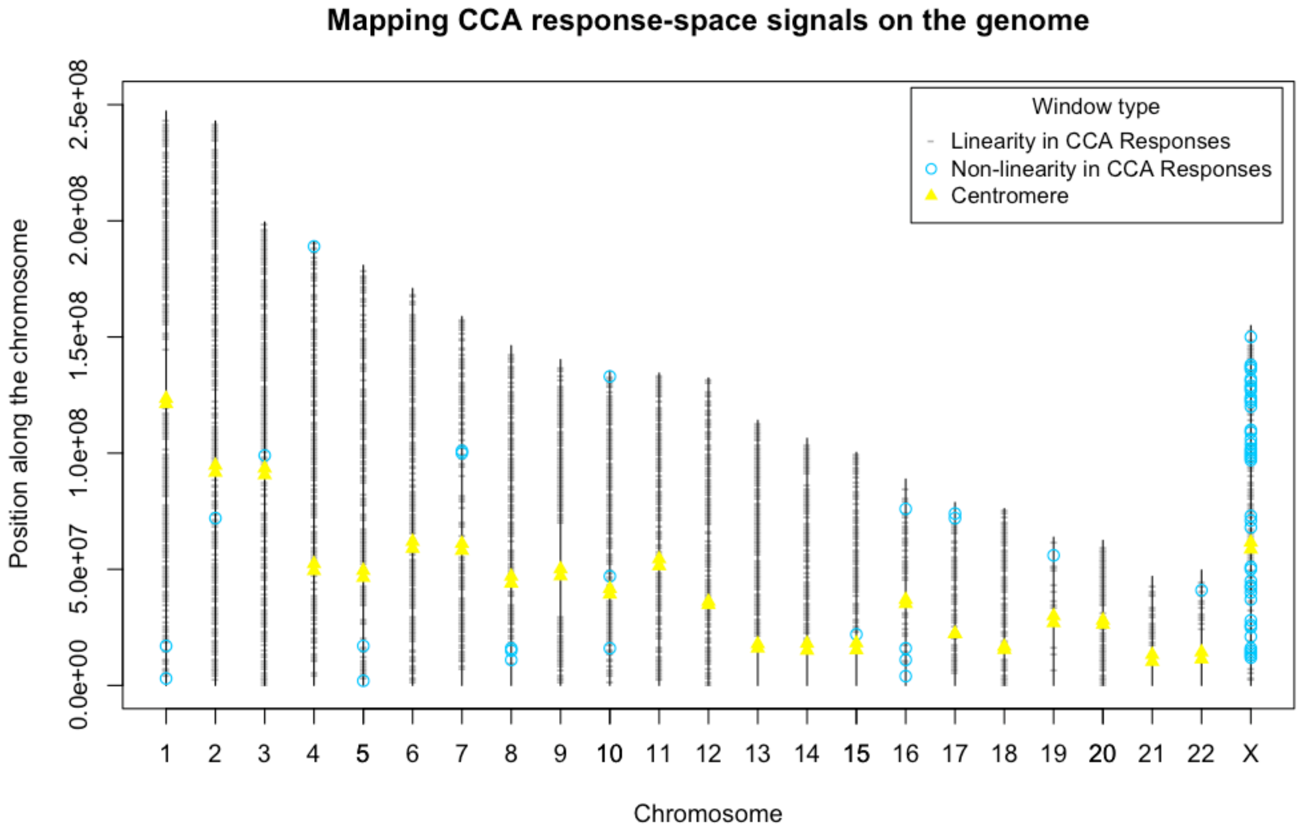
Mapping CCA response-space signals on the genome
Window type
Linearity in CCA Responses
Non-linearity in CCA Responses
Centromere
Position along the chromosome
0.0e+00
5.0e+07
1.0e+08
1.5e+08
2.0e+08
2.5e+08
1
2
3
4
5
6
7
8
9
10
11
12
13
14
15
16
17
18
19
20
21
22
X
Chromosome

C

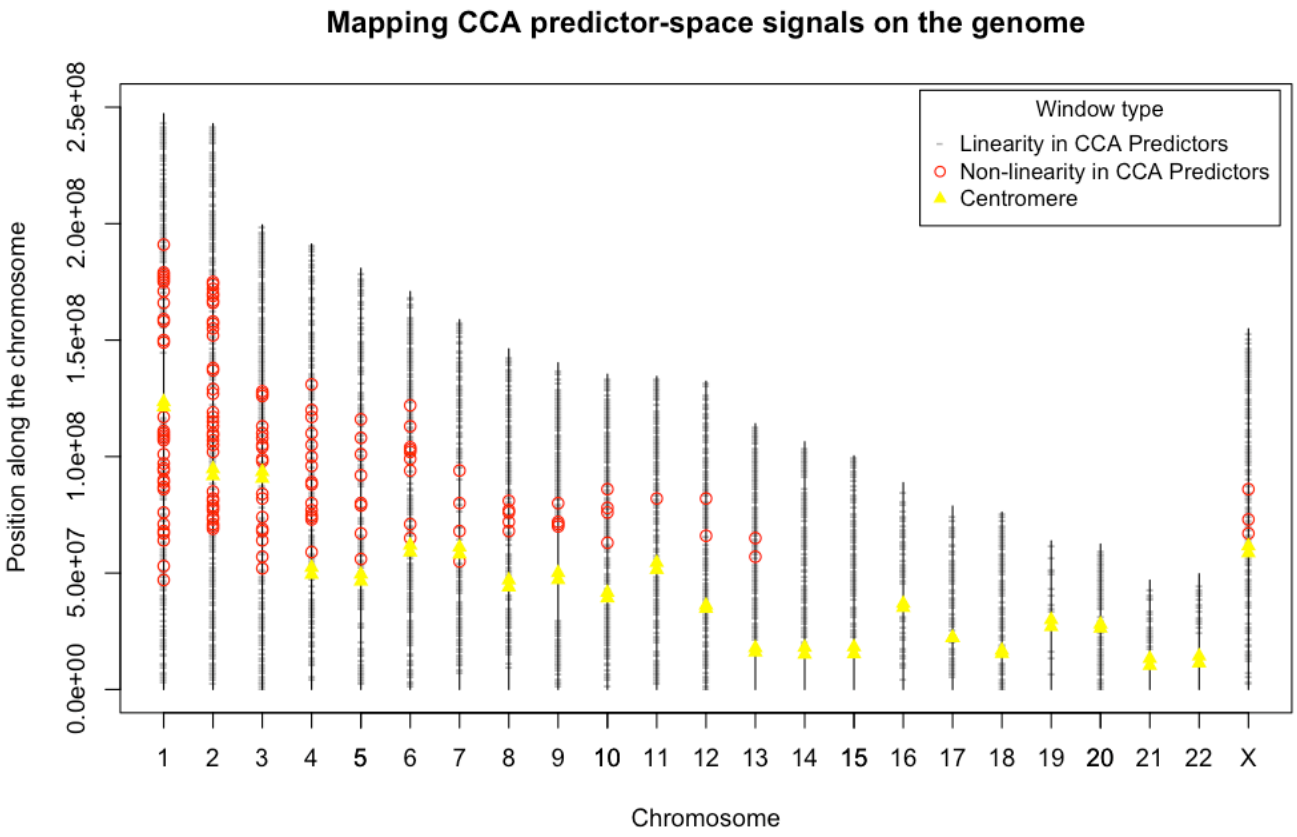
Mapping CCA predictor-space signals on the genome
Window type
Linearity in CCA Predictors
Non-linearity in CCA Predictors
Centromere
Position along the chromosome
0.0e+00
5.0e+07
1.0e+08
1.5e+08
2.0e+08
2.5e+08
1
2
3
4
5
6
7
8
9
10
11
12
13
14
15
16
17
18
19
20
21
22
X
Chromosome

Figure S6.

A

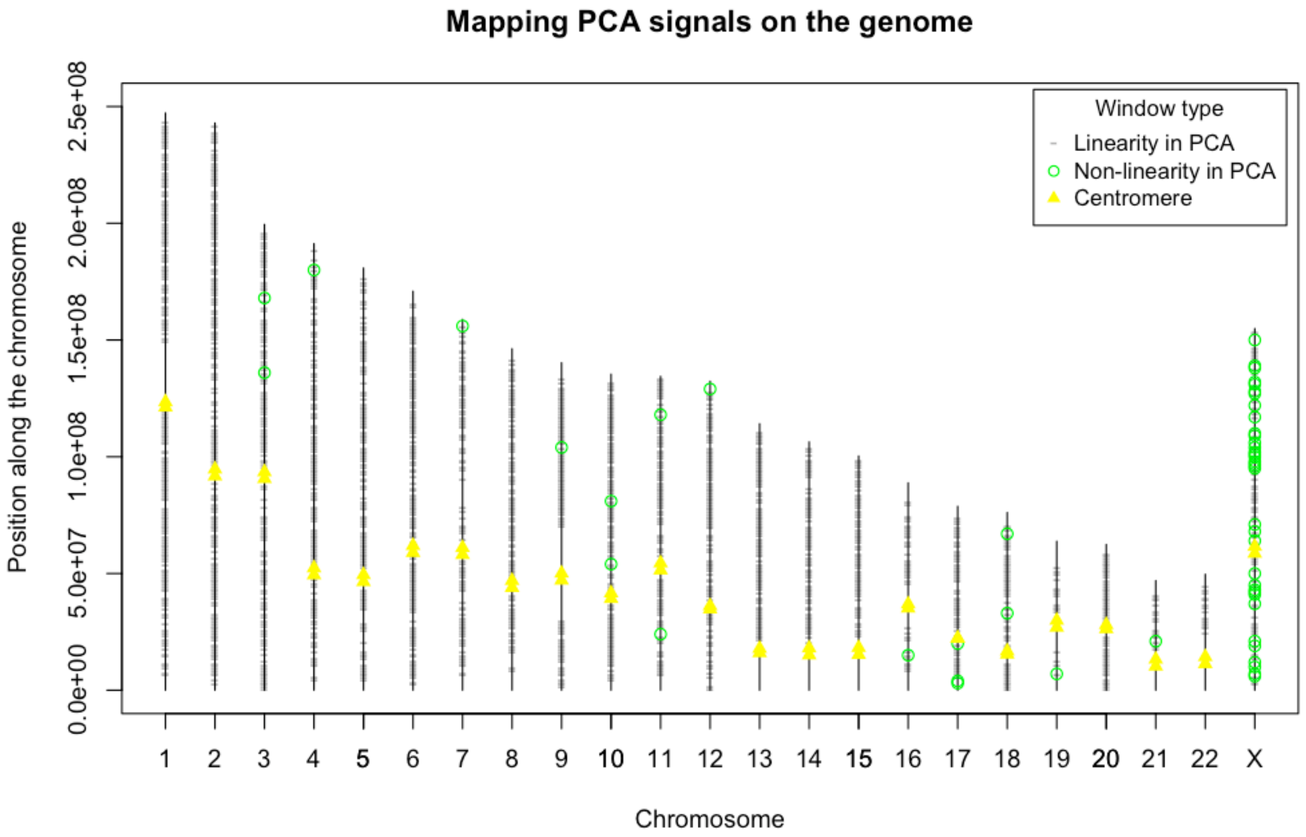

Mapping PCA signals on the genome
Position along the chromosome
0.0e+00
5.0e+07
1.0e+08
1.5e+08
2.0e+08
2.5e+08
Window type
Linearity in PCA
Non-linearity in PCA
Centromere
1
2
3
4
5
6
7
8
9
10
11
12
13
14
15
16
17
18
19
20
21
22
X
Chromosome

B

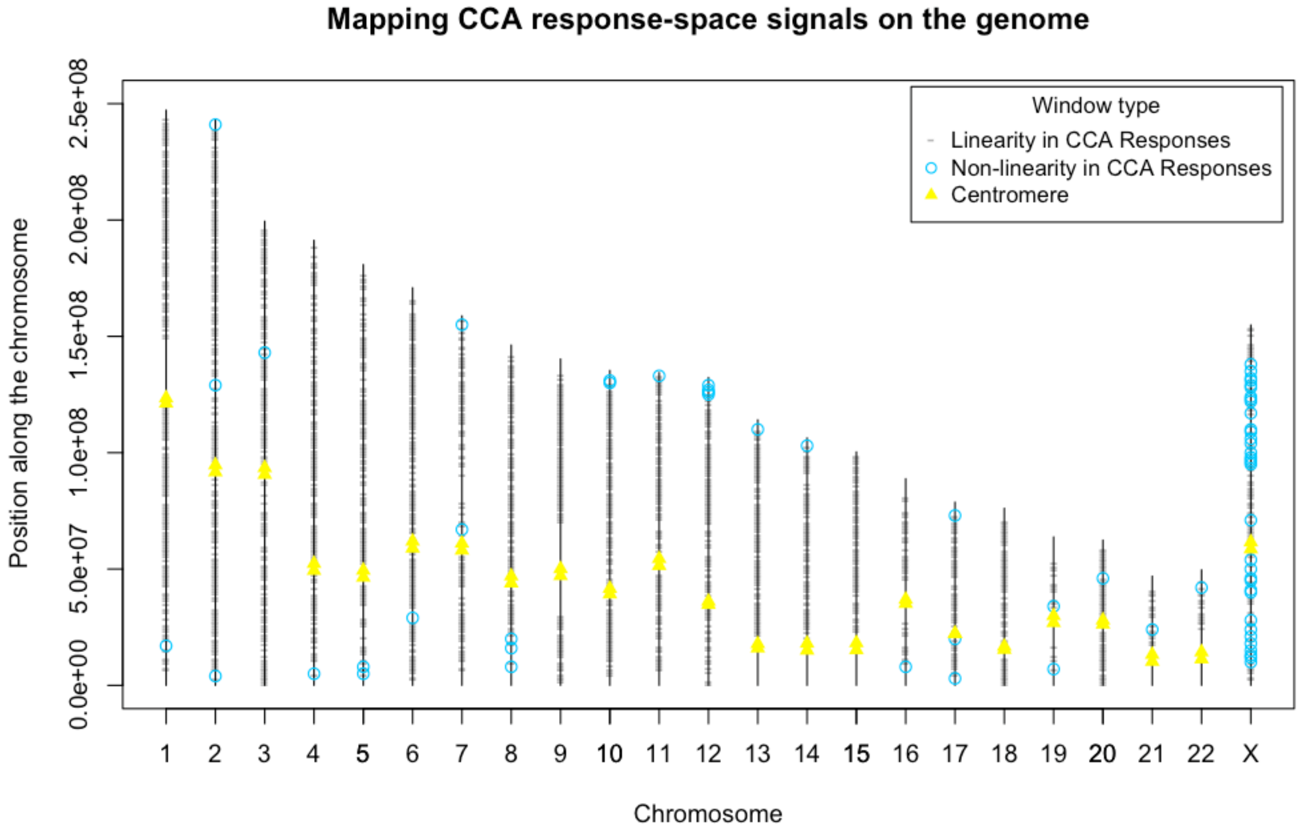
Mapping CCA response-space signals on the genome
Window type
Linearity in CCA Responses
Non-linearity in CCA Responses
Centromere
Position along the chromosome
0.0e+00
5.0e+07
1.0e+08
1.5e+08
2.0e+08
2.5e+08
1
2
3
4
5
6
7
8
9
10
11
12
13
14
15
16
17
18
19
20
21
22
X
Chromosome

C

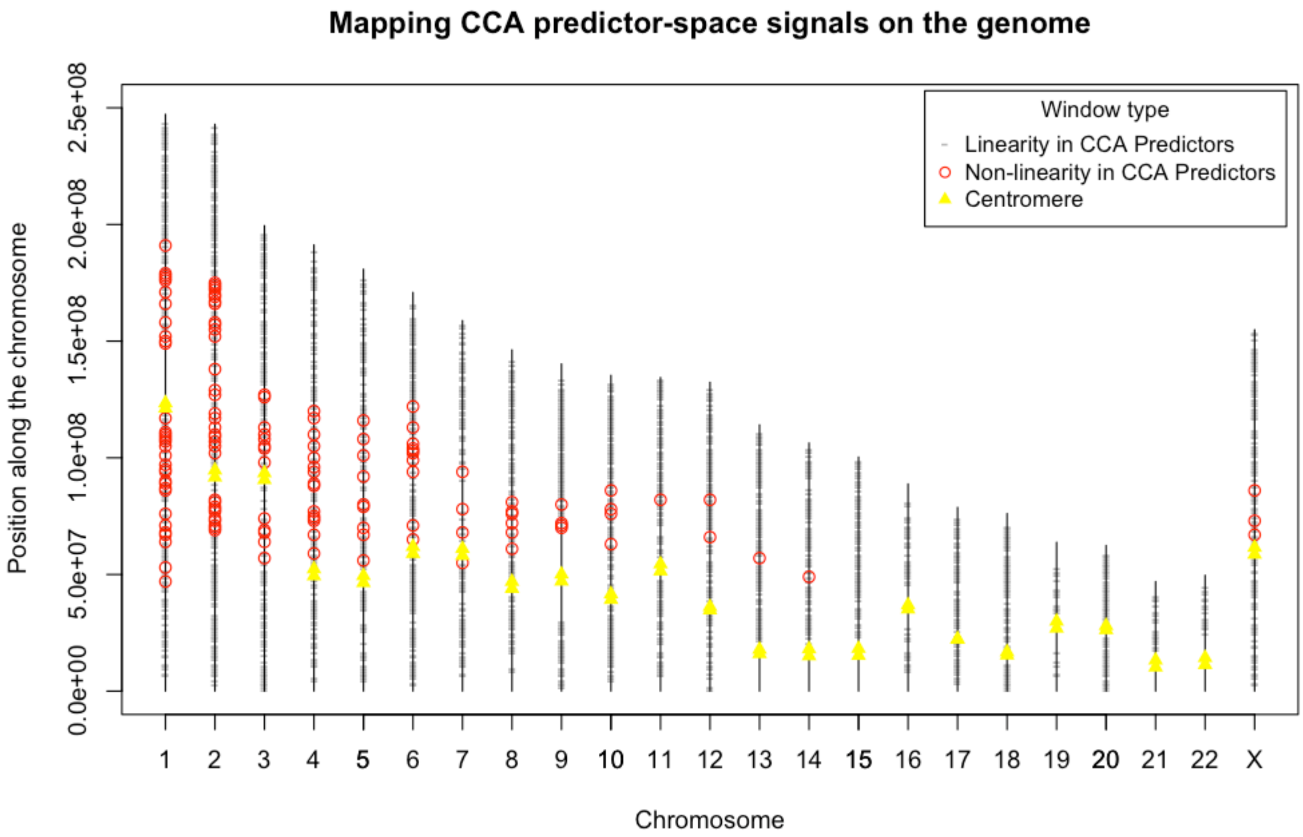
Mapping CCA predictor-space signals on the genome
Window type
Linearity in CCA Predictors
Non-linearity in CCA Predictors
Centromere
Position along the chromosome
0.0e+00
5.0e+07
1.0e+08
1.5e+08
2.0e+08
2.5e+08
1
2
3
4
5
6
7
8
9
10
11
12
13
14
15
16
17
18
19
20
21
22
X
Chromosome

Figure S7.

A

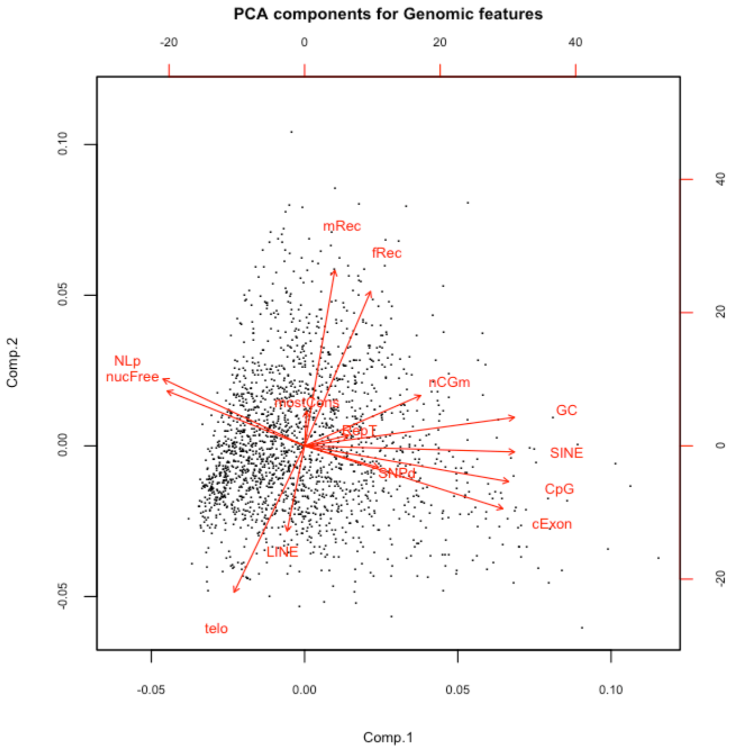
PCA components for Genomic features
-20
0
20
40
0.10
0.05
0.00
-0.05
Comp.2
40
20
0
-20
mRec
fRec
NLp
nucFree
nCGm
GC
SINE
CpG
cExon
SNPd
LINE
telo
-0.05
0.00
0.05
0.10
Comp.1

B

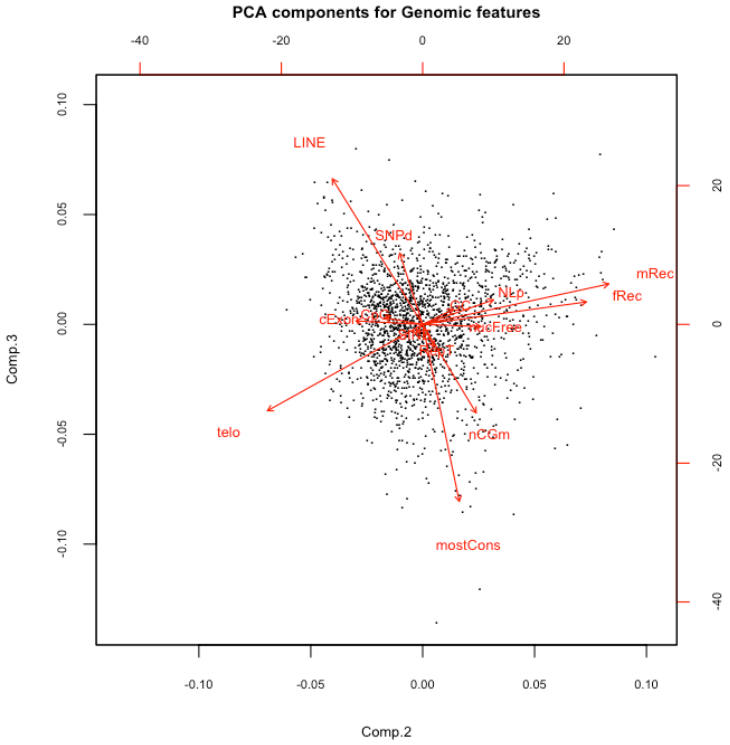
PCA components for Genomic features
-40
-20
0
20
0.10
0.05
0.00
-0.05
-0.10
Comp.3
20
0
-20
-40
LINE
SNPd
NLp
mRec
fRec
telo
nCGm
mostCons
-0.10
-0.05
0.00
0.05
0.10
Comp.2

C

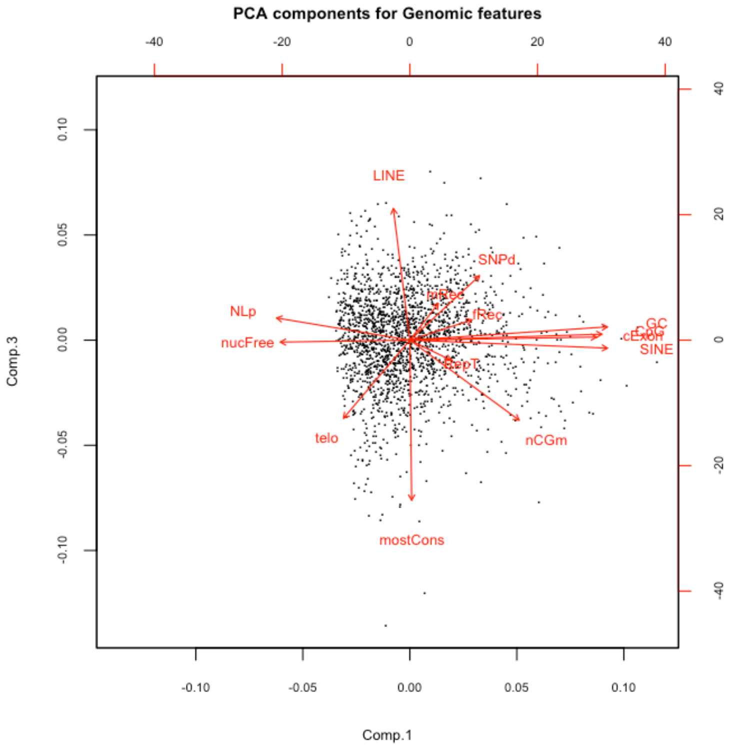
PCA components for Genomic features
-40
-20
0
20
40
0.10
0.05
0.00
-0.05
-0.10
Comp.3
LINE
SNPd
NLp
nucFree
fRec
GC
CpG
cExon
SINE
telo
nCGm
mostCons
-0.10
-0.05
0.00
0.05
0.10
Comp.1

Figure S8.

A

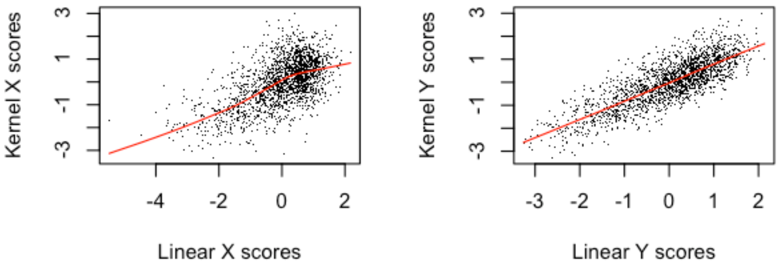

Kernel X scores
3
1
-1
-3
-4
-2
0
2
Linear X scores
Kernel Y scores
3
1
-1
-3
-3
-2
-1
0
1
2
Linear Y scores

B

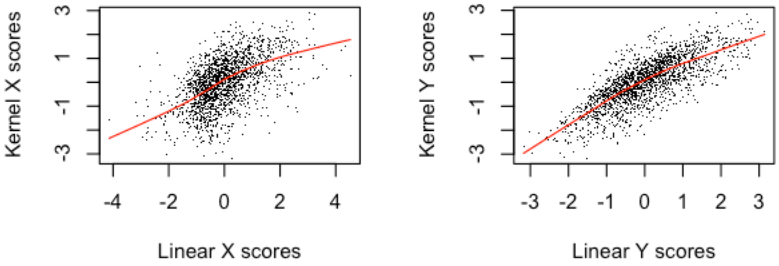

Kernel X scores
3
1
-1
-3
-4
-2
0
2
4
Linear X scores
Kernel Y scores
3
1
-1
-3
-3
-2
-1
0
1
2
3
Linear Y scores

Figure S9.

A.

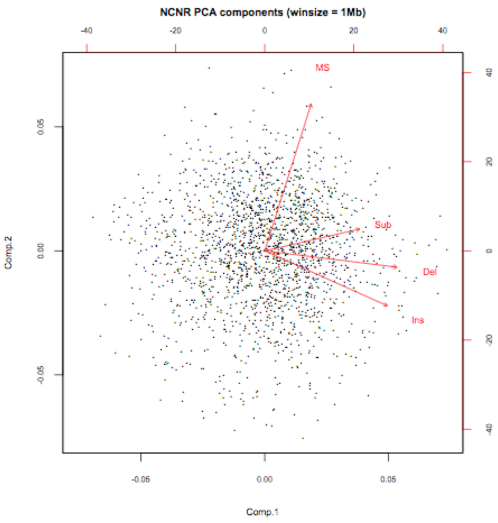
NCNR PCA components (winsize = 1Mb)
MS
Sub
Del
Ins
Comp.1
Comp.2

B.

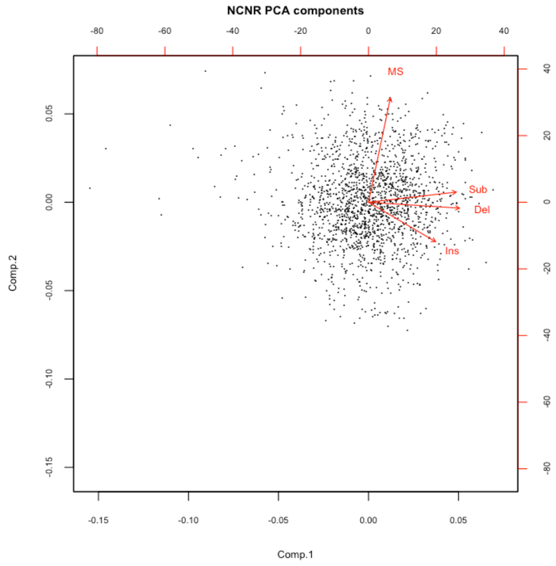
NCNR PCA components
MS
Sub
Del
Ins
Comp.1
Comp.2

Figure S10.

A.

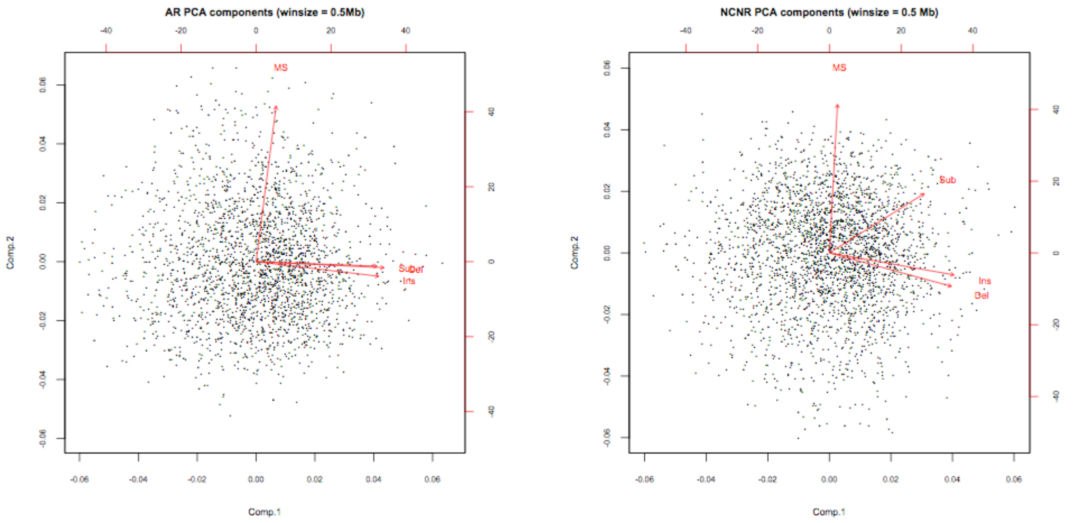

B.

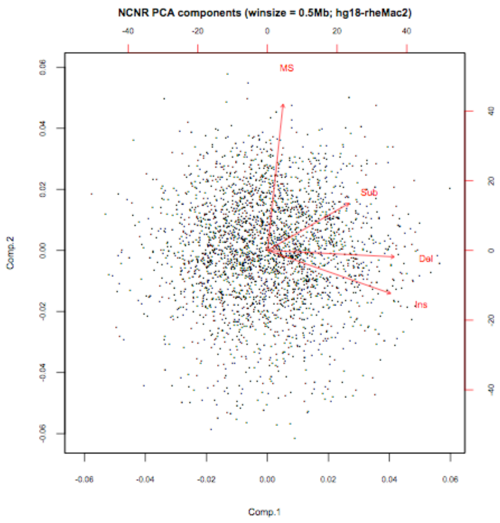

C.

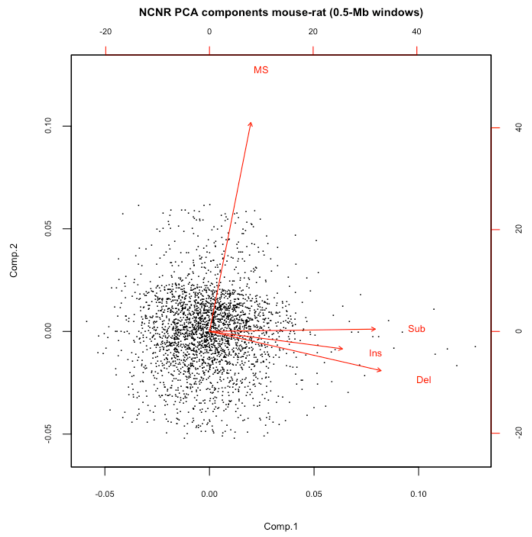

Figure S11.

A.

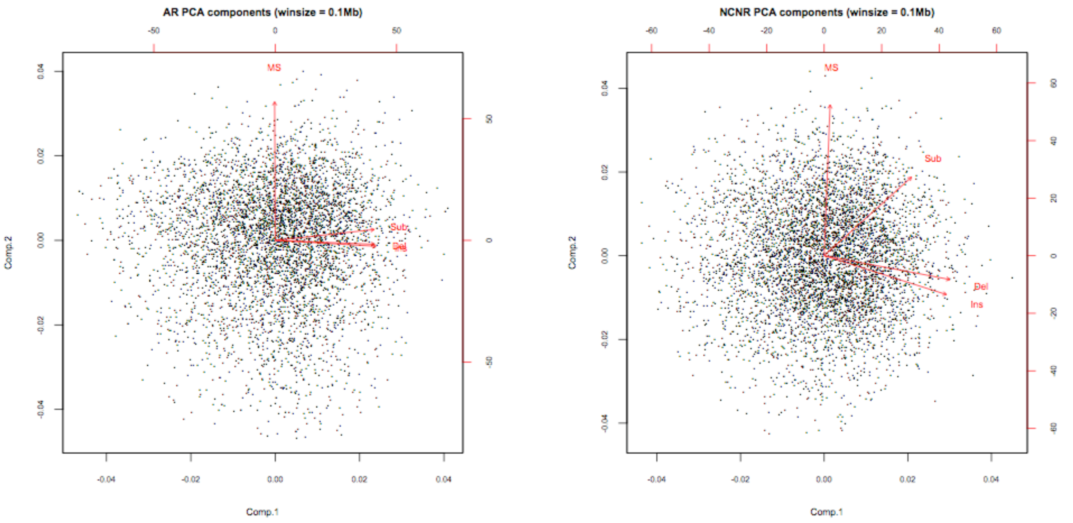

B.

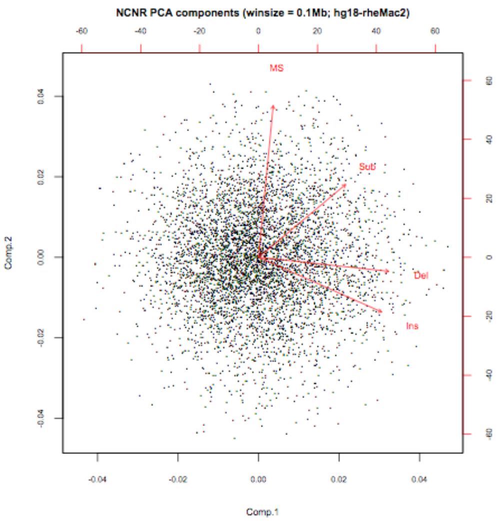

C.

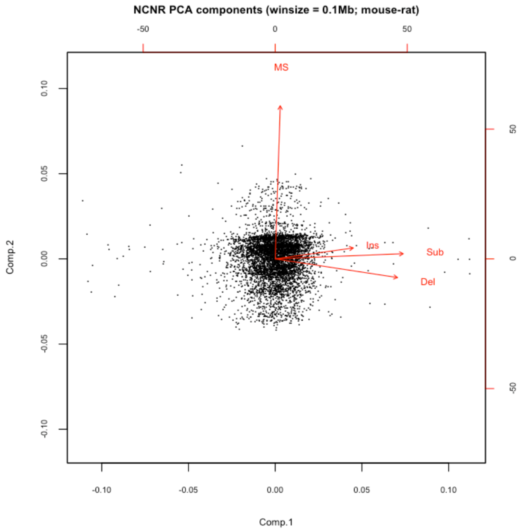

Figure S12.

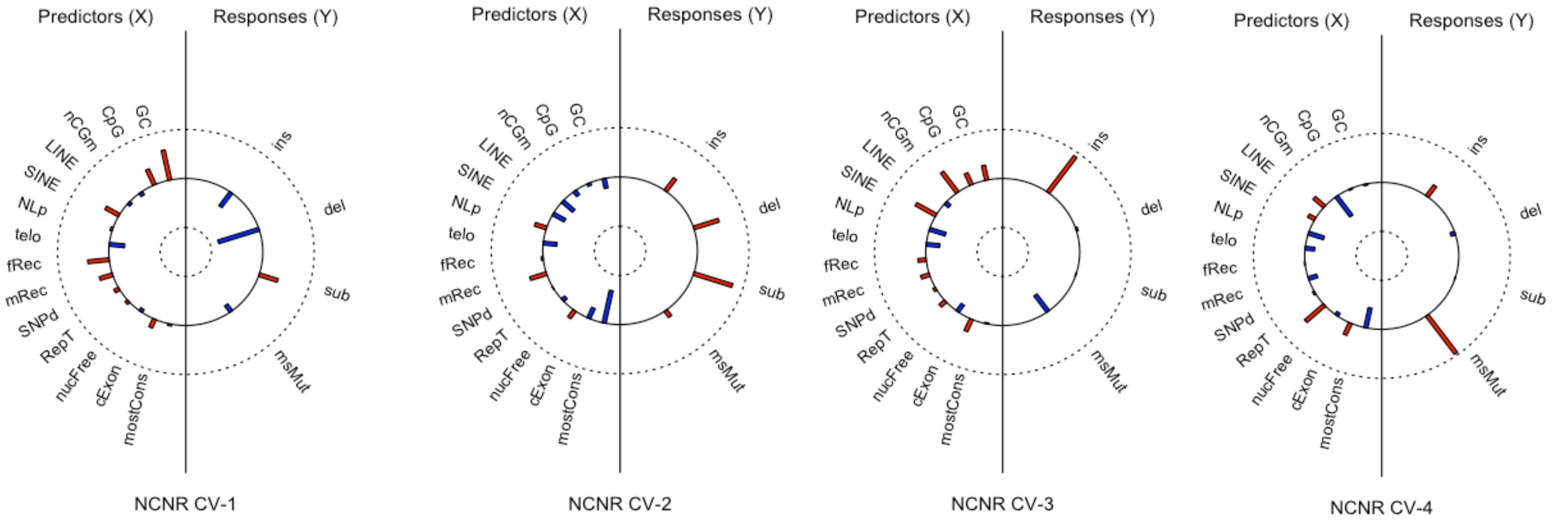
Predictors (X)
Responses (Y)
GC
CpG
nCGm
LINE
SINE
NLp
telo
fRec
mRec
SNPd
RepT
nucFree
cExon
mostCons
ins
del
sub
msMut
NCNR CV-1
Predictors (X)
Responses (Y)
GC
CpG
nCGm
LINE
SINE
NLp
telo
fRec
mRec
SNPd
RepT
nucFree
cExon
mostCons
ins
del
sub
msMut
NCNR CV-2
Predictors (X)
Responses (Y)
GC
CpG
nCGm
LINE
SINE
NLp
telo
fRec
mRec
SNPd
RepT
nucFree
cExon
mostCons
ins
del
sub
msMut
NCNR CV-3
Predictors (X)
Responses (Y)
GC
CpG
nCGm
LINE
SINE
NLp
telo
fRec
mRec
SNPd
RepT
nucFree
cExon
mostCons
ins
del
sub
msMut
NCNR CV-4

Figure S13. A

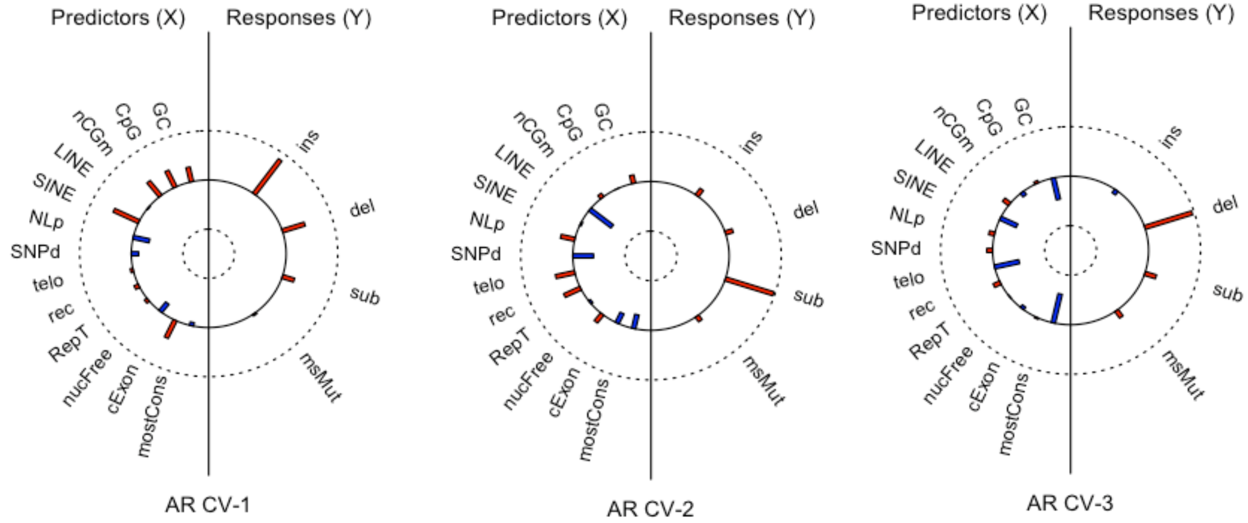

B

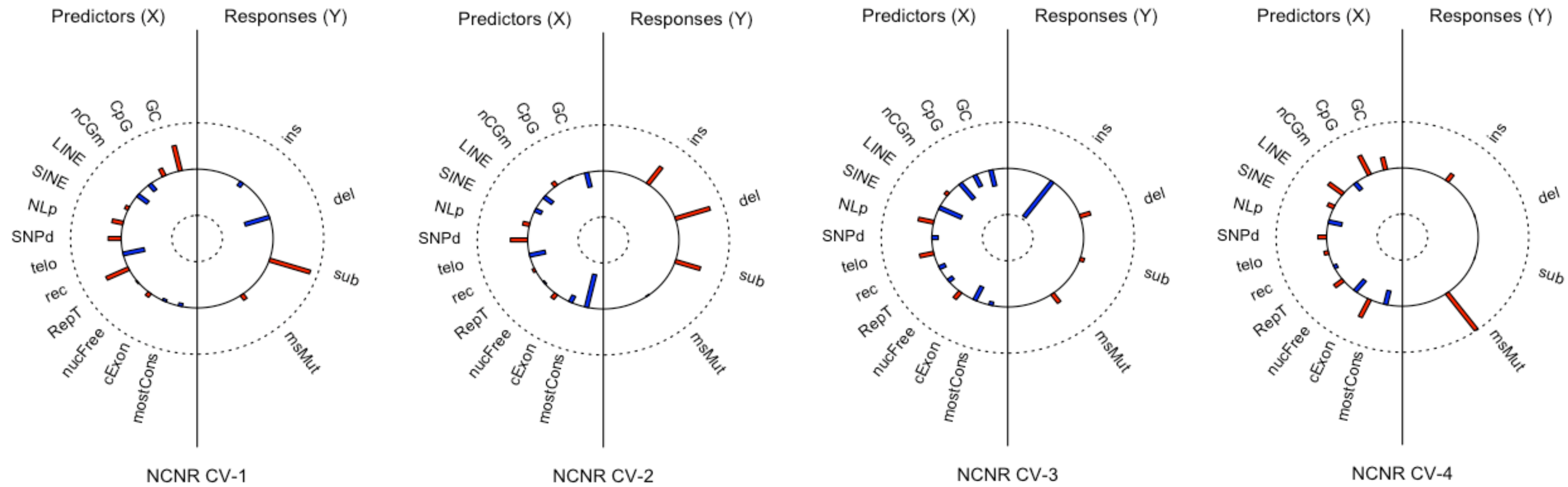

Figure S14.

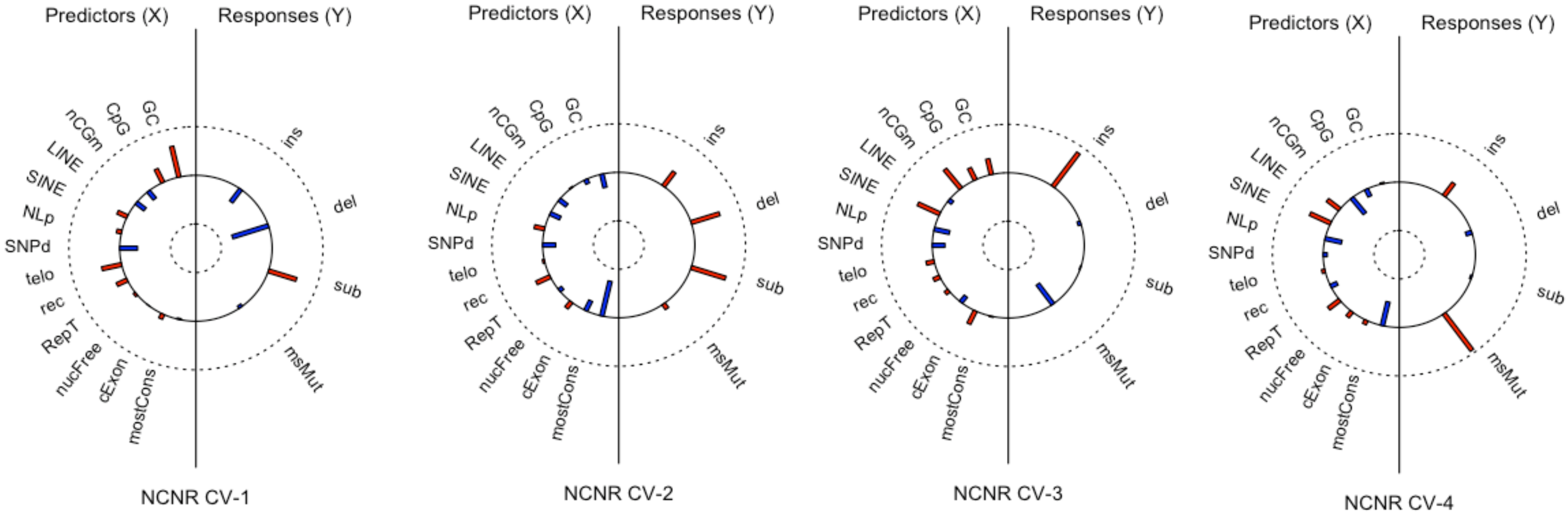
Predictors (X)
Responses (Y)
GC
CpG
nCGm
LINE
SINE
NLp
SNPd
telo
rec
RepT
nucFree
cExon
mostCons
ins
del
sub
msMut
NCNR CV-1
NCNR CV-2
NCNR CV-3
NCNR CV-4

Figure S15. A

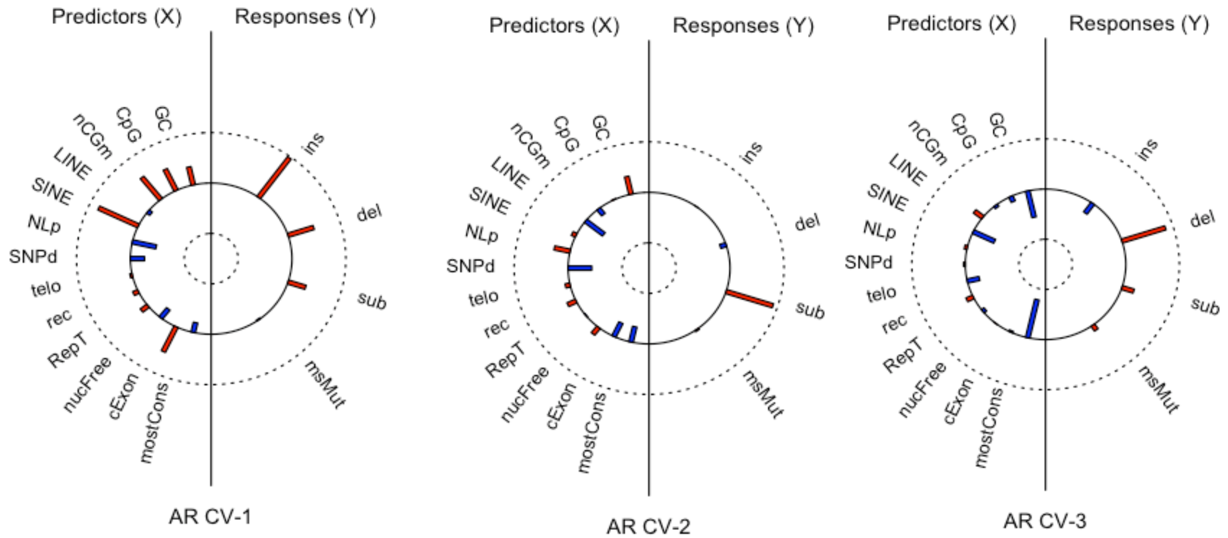

B

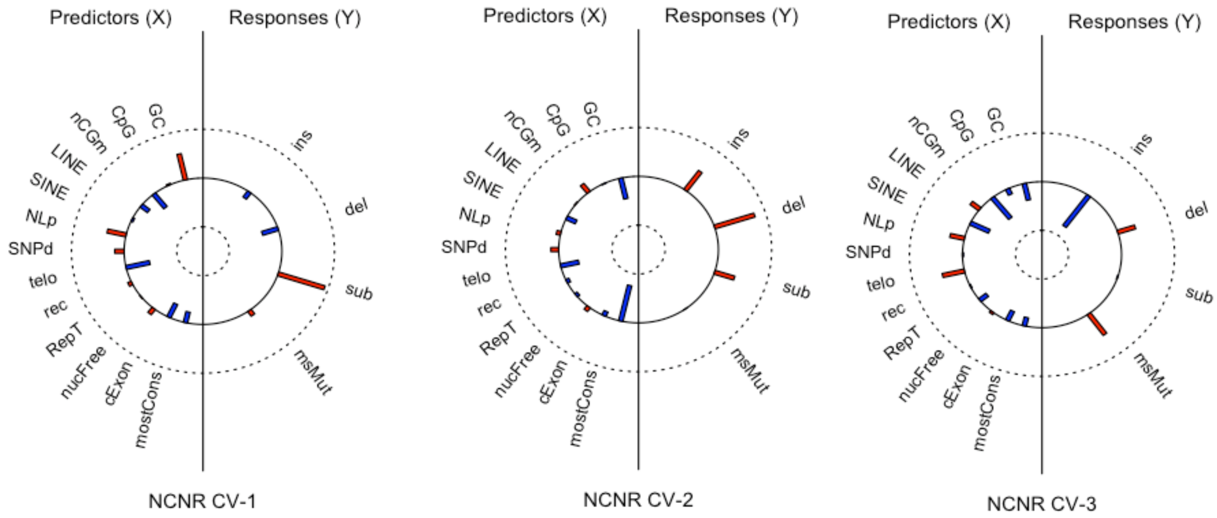

Figure S16.

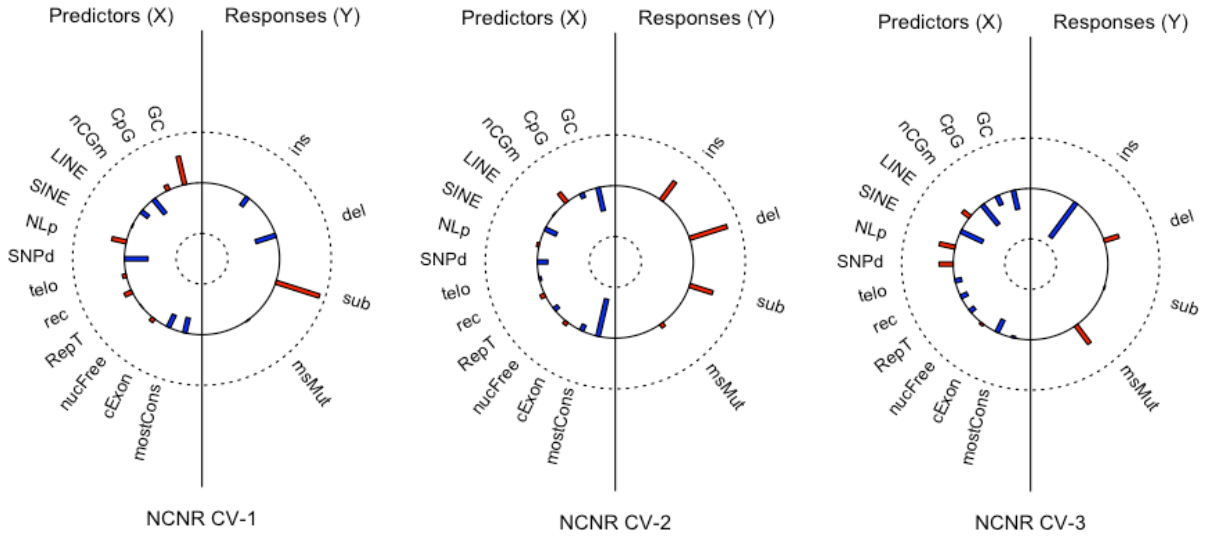
Predictors (X)
Responses (Y)
GC
CpG
nCGm
LINE
SINE
NLp
SNPd
telo
rec
RepT
nucFree
cExon
mostCons
ins
del
sub
msMut
NCNR CV-1
NCNR CV-2
NCNR CV-3

Figure S17. A

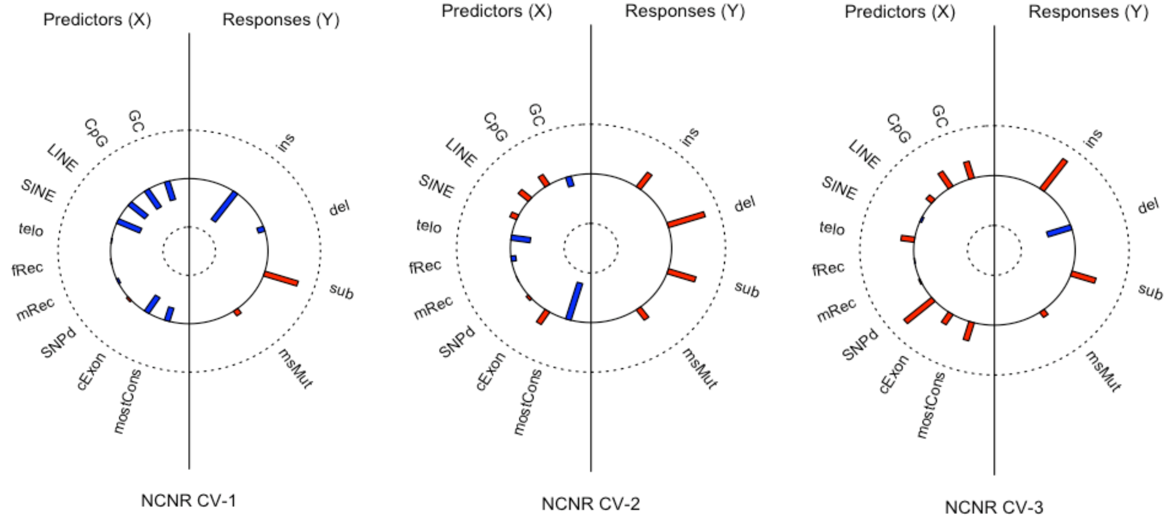

B.

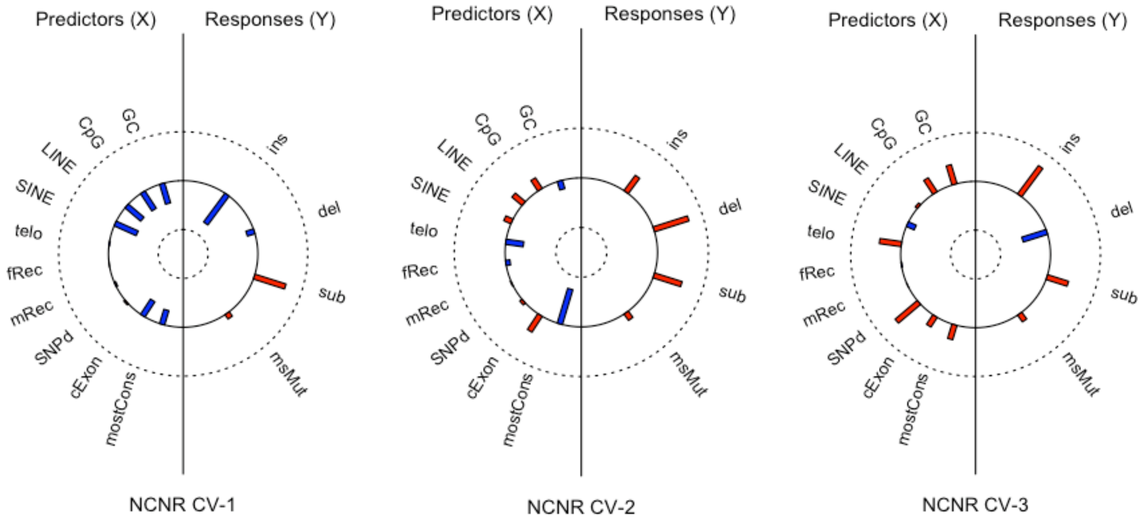

C.

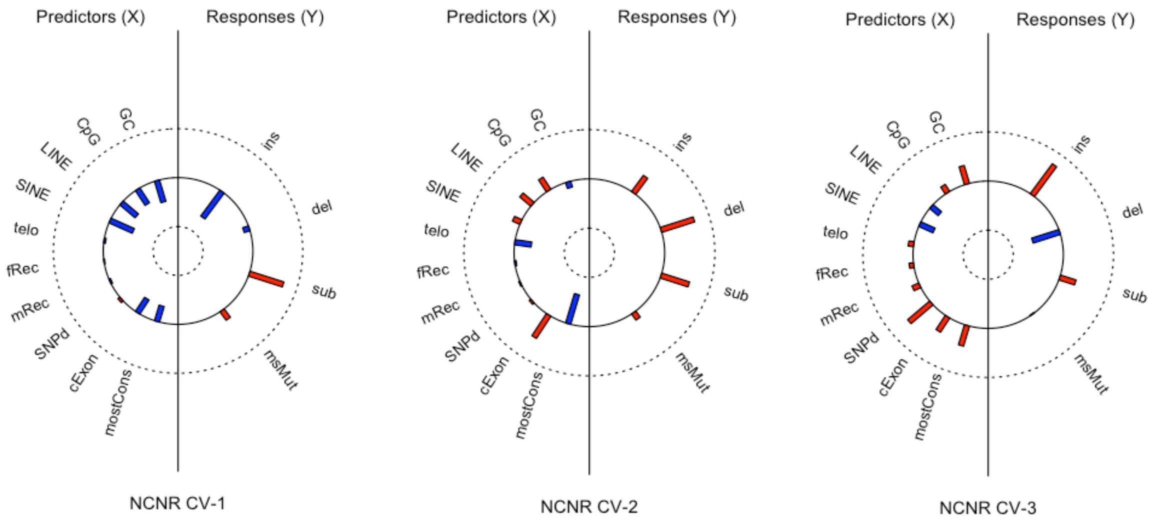
Predictors (X)
Responses (Y)
GC
CpG
LINE
SINE
telo
fRec
mRec
SNPd
cExon
mostCons
ins
del
sub
msMut
NCNR CV-1
NCNR CV-2
NCNR CV-3

Table S1. PCA on mutation rates: Summary

Component	AR		NCNR	
	Variance	Proportion of variance explained	Variance	Proportion of variance explained
1	2.1525	0.5381	1.8325	0.4581
2	0.9972	0.2493	1.0250	0.2563
3	0.4912	0.1229	0.7056	0.1764
4	0.3585	0.0896	0.4368	0.1092

Variance and proportion of variance explained by the four principal components for AR and NCNR autosomal regions. The first two components have variance greater than one, and account for 79% (AR) and 71% (NCNR) of the total variance.

Table S2. PCA on mutation rates: Loadings

Sub-genome	AR		NCNR	
**Variable/Component**	**1**	**2**	**1**	**2**
Insertion rate	0.569	0.0	0.619	0.0
Deletion rate	0.598	0.0	0.610	0.0
Nucleotide substitution rate	0.562	0.0	0.493	0.258
Microsatellite mutability	0.0	0.997	0.0	0.959

Loadings for the first and second principal components in the AR and NCNR sub-genomes.

Table S3. Regression results for first kernel principal component scores versus first two linear principal component scores

**Sub-genome**	**AR**		**NCNR**	
**Predictors**	***t*-value**	***p*-value**	***t*-value**	***p*-value**
PCA component 1	82.00	< 2e-16	77.56	<2e-16
PCA component 2	-6.17	9e-10	-14.89	<2e-16
Multiple R^2	0.7697		0.7618	
Adjusted R^2	0.7694		0.7616	

Table S4. PCA on genomic landscape variables: Summary

Component	Variance	Proportion of variance explained
1	4.0662	0.2904
2	1.7453	0.1247
3	1.2846	0.0918
4	1.0697	0.0764
5	0.9732	0.0652
6	0.8654	0.0618
7	0.8089	0.0578
8	0.7349	0.0525
9	0.6750	0.0482
10	0.5748	0.0411
11	0.5342	0.0382
12	0.2712	0.0194
13	0.2431	0.0174
14	0.1534	0.0109

Variance and proportion of variance explained by the fourteen principal components computed on genomic landscape variables. The first three components have variance greater than one, and account for 50% of the total variance.

Table S5. PCA on genomic landscape variables: Loadings

**Variable/Component**	**1**	**2**	**3**
GC	-0.436	0.0	0.0
CpG	-0.399	0.0	-0.132
nCGm	-0.208	0.137	0.456
LINE	0.0	-0.259	-0.157
SINE	-0.408	0.0	0.199
NLp	0.288	0.273	0.0
Telo	0.190	-0.457	0.249
fRec	-0.138	0.449	0.0
mRec	-0.142	0.538	-0.207
SNPd	-0.164	-0.109	-0.418
RepT	-0.102	0.0	0.0
nucFree	0.266	0.220	0.0
cExon	-0.407	-0.230	0.0
mostCons	0.0	0.0	0.640

Loadings for the first three principal components computed on genomic landscape variables. GC – GC content, CpG – number of CpG islands, nCGm – number of methyl-cytosines in non-CpG context, LINE – number of LINE elements, SINE – number of SINE elements, NLp – number of nuclear lamina associated regions, Telo – distance to the telomere, fRec and mRec – female and male recombination rates respectively, SNPd – SNP density, RepT – replication time, nucFree – density of nucleosome-free regions, cExon – coverage by coding exons, mostCons – coverage by most conserved elements.

Table S6. CCA correlations and *p*-values

**Sub-genome**	**AR**				**NCNR**			
**Component**	**1**	**2**	**3**	**4**	**1**	**2**	**3**	**4**
Correlation	0.7338	0.5336	0.3287	0.0534	0.6955	0.5043	0.3906	0.1043
p-value	<2e-16	<2e-16	<2e-16	0.7637	<2.2e-16	<2.2e-16	<2.2e-16	0.0116

Correlations and *p*-values for canonical correlation component pairs in the AR and NCNR sub-genomes. Correlations are computed between the predictor and response canonical variates (CVs) in each pair, and *p*-values represent their statistical significance.

Table S7. Regression results for first kernel canonical component scores versus significant linear canonical component scores

**Sub-genome (co-variate)**	**AR (Genomic features)**		**NCNR (Genomic features)**		**AR (Mutation rates)**		**NCNR (Mutation rates)**	
**Predictors**	***t*-value**	***p*-value**	***t*-value**	**p-value**	***t*-value**	***p*-value**	***t*-value**	***p*-value**
CCA component 1	2.00	0.045	-3.44	0.0006	1154	< 2e-16	5e6	<2e-16
CCA component 2	-4.06	5e-05	3.83	0.0001	-2750	< 2e-16	-3e6	<2e-16
CCA component 3	-0.935	0.35	-1.54	0.1231	1213	< 2e-16	5e6	<2e-16
CCA component 4	NA	NA	-0.19	0.8490	NA	NA	-3e5	<2e-16
Multiple R^2	0.0105		0.0146		1.0		1.0	
Adjusted R^2	0.0090		0.0126		1.0		1.0	

NA: Not included in the regression because the component was not significant.

Table S8. Proportion of the AR and NCNR sub-genomes covered by the 8-way Multiz alignments of human, chimpanzee, orangutan, rhesus macaque, marmoset, mouse, opossum, and platypus (obtained from the multiz8way table of the UCSC Genome Browser using the ponAbe2 build of Orangutan).

Sub-genome	Species	Basepair coverage (%)
**NCNR**	Human	76.59
	Orangutan	76.14
	Macaque	70.56
**AR**	Human	88.64
	Orangutan	88.02
	Macaque	75.09

Table S9. Number of windows before and after outlier filtering

Phylogenetic branch	Genomic scale	AR		NCNR	
		Before	After	Before	After
**Human-Orangutan**	1-Mb	2264	2027	2273	1953
	0.5-Mb	3018	2736	3575	3159
	0.1-Mb	5639	5101	6400	5628
**Human-Macaque**	1-Mb	2275	2055	2274	2013
	0.5-Mb	3582	3226	3582	3183
	0.1-Mb	6660	5936	6252	5452
**Mouse-Rat**	1-Mb	NA	NA	2143	1754
	0.5-Mb	NA	NA	3454	2763
	0.1-Mb	NA	NA	6252	5452
